# Lysophosphatidic Acid and Several Neurotransmitters Converge on Rho-Kinase 2 Signaling to Manage Motoneuron Excitability

**DOI:** 10.3389/fnmol.2021.788039

**Published:** 2021-12-06

**Authors:** Victoria García-Morales, Ángela Gento-Caro, Federico Portillo, Fernando Montero, David González-Forero, Bernardo Moreno-López

**Affiliations:** ^1^GRUpo de NEuroDEgeneración y NeurorREparación (GRUNEDERE), Área de Fisiología, Facultad de Medicina, Universidad de Cádiz, Cádiz, Spain; ^2^Instituto de Investigación e Innovación Biomédica de Cádiz (INiBICA), Cádiz, Spain

**Keywords:** intrinsic membrane excitability, background potassium channels, lysophosphatidic acid (LPA), serotonin, thyrotropin-releasing hormone (TRH)

## Abstract

Intrinsic membrane excitability (IME) sets up neuronal responsiveness to synaptic drive. Several neurotransmitters and neuromodulators, acting through G-protein-coupled receptors (GPCRs), fine-tune motoneuron (MN) IME by modulating background K^+^ channels TASK1. However, intracellular partners linking GPCRs to TASK1 modulation are not yet well-known. We hypothesized that isoform 2 of rho-kinase (ROCK2), acting as downstream GPCRs, mediates adjustment of MN IME via TASK1. Electrophysiological recordings were performed in hypoglossal MNs (HMNs) obtained from adult and neonatal rats, neonatal knockout mice for TASK1 (*task1*^–/–^) and TASK3 (*task3*^–/–^, the another highly expressed TASK subunit in MNs), and primary cultures of embryonic spinal cord MNs (SMNs). Small-interfering RNA (siRNA) technology was also used to knockdown either ROCK1 or ROCK2. Furthermore, ROCK activity assays were performed to evaluate the ability of various physiological GPCR ligands to stimulate ROCK. Microiontophoretically applied H1152, a ROCK inhibitor, and siRNA-induced ROCK2 knockdown both depressed AMPAergic, inspiratory-related discharge activity of adult HMNs *in vivo*, which mainly express the ROCK2 isoform. In brainstem slices, intracellular constitutively active ROCK2 (aROCK2) led to H1152-sensitive HMN hyper-excitability. The aROCK2 inhibited pH-sensitive and TASK1-mediated currents in SMNs. Conclusively, aROCK2 increased IME in *task3*^–/–^, but not in *task1*^–/–^ HMNs. MN IME was also augmented by the physiological neuromodulator lysophosphatidic acid (LPA) through a mechanism entailing G_αi/o_-protein stimulation, ROCK2, but not ROCK1, activity and TASK1 inhibition. Finally, two neurotransmitters, TRH, and 5-HT, which are both known to increase MN IME by TASK1 inhibition, stimulated ROCK2, and depressed background resting currents via G_αq_/ROCK2 signaling. These outcomes suggest that LPA and several neurotransmitters impact MN IME via G_αi/o_/G_αq_-protein-coupled receptors, downstream ROCK2 activation, and subsequent inhibition of TASK1 channels.

## Introduction

Intrinsic membrane excitability (IME) sets up neuronal responsiveness to afferent activity. Neuronal IME depends on both passive and active membrane properties. Passive membrane properties, such as resting membrane potential (Vm) and membrane resistance, are mainly determined by the density of background K^+^ channels that are leaky at rest. On the other hand, active properties mainly rely on the state of voltage- and ligand-gated ion channels. In this framework, whilst fine-tuning of IME manages physiological neuronal responses, disturbance of this accurate regulation might delimit the cutting edge behind which pathological events arise leading to neurodegeneration. Accordingly, alterations in IME turned specific neuronal pools into predominantly susceptible in a number of neurodegenerative disorders (Saxena and Caroni, 2011; Roselli and Caroni, 2015). Since IME malleability defines plastic skill of neuronal networks that affects neuronal vulnerability to degeneration, knowledge of the mechanisms that control this neuronal property is central for understanding its relevance in both physiologic and pathologic states.

The potassium channel family KCNK of two-pore-domain K^+^ channels makes a leading contribution to the resting background conductance and strongly affects IME in mammalian excitable cells (Bayliss et al., 2003). Furthermore, KCNK channels have a potential therapeutic impact in cancer, pain, inflammation, ischemia, depression, and epilepsy (Bayliss and Barrett, 2008; Bittner et al., 2010). Specifically, the pH-sensitive subunits, TWIK-related acid-sensitive K^+^-1 (TASK1) and TASK3, are widely co-expressed throughout the brain, with particularly high expression levels in motoneurons (MNs) (Talley et al., 2001). Consequently, it is well-known that MN IME is mainly defined by TASK1/3 heterodimers, along with TASK1/1 and TASK3/3 homodimers (Talley et al., 2001; Berg et al., 2004). TASK currents can be modulated by multiple neurotransmitter systems, including those associated with awakening and alertness states, which likely serves to couple neuronal responsiveness to afferent drive and behavioral status (McCormick and Bal, 1997; Talley et al., 2000; Bayliss et al., 2003). Several neurotransmitters acting on G-protein-coupled receptors (GPCRs) evoke slow excitation in MNs by full inhibition of TASK1 via a mechanism comprising a direct interaction of G_αq_ or a closely associated intermediary with a channel (Talley et al., 2000; Chen et al., 2006). Finally, impaired expression of TASK1 lies behind increased IME and vulnerability to excitotoxic degeneration of MNs in several pathological conditions (García-Morales et al., 2019). Hence, identifying factors which impact MN IME by modulating TASK1 channels has basic and clinical significance.

**FIGURE 1:**
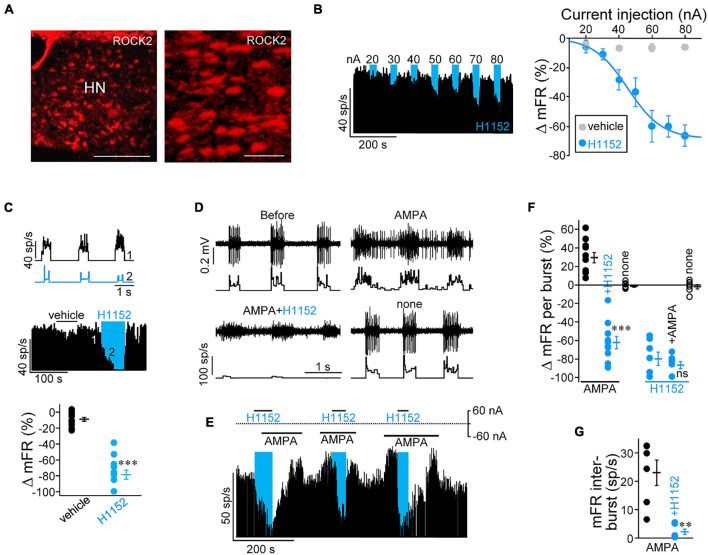
The rho-associated coiled-coil-containing kinases (ROCK) inhibitor, H1152, strongly reduces inspiratory-related and (RS)-α-amino-3-hydroxy-5-methyl-4-isoxazolepropionic acid (AMPA)-induced discharge activity of hypoglossal motoneurons (HMNs) *in vivo*. **(A)** Low-magnification epifluorescence images showing the overall expression pattern of isoform 2 of rho-kinase (ROCK2) in the hypoglossal motor nucleus (HN) of an adult rat. Scale bars: left, 300 μm; right, 75 μm. **(B)** Left, time course of the mean firing rate (mFR, in sp/s) per burst obtained from the unitary recording of a HMN during microiontophoretic administration (30 s on, 60 s off) of H1152 (20 mM) at progressively increasing ejection currents. Right, mean current-response curves illustrating the effects of microiontophoretically-administered H1152 (blue symbols) or vehicle (black symbols) on the mFR per burst in the inspiratory-related discharge of HMNs. Vehicle, *n* = 7 HMNs; H1152, *n* = 6 HMNs. **(C)** Top, instantaneous firing rate (sp/s) of a HMN in response to sequential microiontophoretic administration (+80 nA, 60 s) of vehicle (1) and H1152 (2). Middle, time course of changes in mFR per burst for the same HMN under the previously described application protocol. Bottom, maximal change in mFR induced by current application through the indicated solutions. *n* = 12 HMNs. **(D)** Extracellularly recorded spike discharge (top) for a HMN displaying the characteristic inspiratory-related burst of action potentials and the histogram of the instantaneous firing rate (sp/s, bottom) before (retention current applied, ±5 nA), and after sequential microiontophoretic ejection first of AMPA (400 μM, –60 nA), then of AMPA (–60 nA) plus H1152 (+ 60 nA) from different barrels, and finally without ejection of drug solution (retention current applied, ± 5 nA). **(E)** As in **(B)**, but combining different sequences of ejection for AMPA and H1152. **(F,G)** Maximal changes in mFR per burst (**F**, *n* ≥ 7 HMNs) and in inter-burst intervals (**G**, *n* = 6 HMNs) under ejection of stated drugs. Blue boxes indicate intervals during which H1152 was applied. Error bars, SEM. ^∗∗^*p* < 0.01, ^∗∗∗^*p* < 0.001; ns, not significant; by paired Student’s *t*-test.

Rho-associated coiled-coil-containing kinases (ROCK), originally identified as major downstream effectors of the small GTPase RhoA, are potential candidates to mediate the regulatory action on MN IME of a number of neuromodulators and neurotransmitters. The two identified mammalian ROCK homologs, ROCK1 (ROCKβ) and ROCK2 (ROCKα), belong to the AGC family of serine/threonine kinases (protein kinases A, G, and C). Although both isotypes are ubiquitously expressed, ROCK1 is preferentially expressed in kidney, liver, spleen, and testis, while ROCK2 is enriched in brain, heart, lung, and skeletal muscle (Hartmann et al., 2015; Liu et al., 2018; Seccia et al., 2020). RhoA/ROCK signaling has been shown to exert a regulatory role of a broad array of cellular processes such as cytoskeletal organization by controlling F-actin stabilization, actomyosin contraction, microtubule assembly, cell adhesion and motility, proliferation and apoptosis, and remodeling of the extracellular matrix or smooth muscle contraction (Hartmann et al., 2015; Liu et al., 2018; Seccia et al., 2020). However, little attention has been paid on the role of ROCK on cell excitability by its direct influence on intrinsic membrane properties in spite of its known regulatory action on several ionic channels (Li et al., 2002; Piccoli et al., 2004; Staruschenko et al., 2004; Iftinca et al., 2007; Seyler et al., 2012; García-Morales et al., 2019). In particular, ROCK modulates TASK1-mediated currents through short and long-term mechanisms. For instance, ROCK inhibits these subunits by direct channeling of phosphorylation at Ser^393^ in human pulmonary artery smooth muscle cells during endothelin-1-induced vasoconstriction (Seyler et al., 2012). A ROCK-dependent mechanism that impairs traffic of TASK1 to the plasma membrane has also been shown to underlie downregulation of TASK currents following long-term exposure to nitric oxide at pathological concentrations (García-Morales et al., 2019). Furthermore, lysophosphatidic acid (LPA) signaling, an upstream activator of ROCK, increases MN IME in a TASK1-dependent manner (Gento-Caro et al., 2021a,b). Finally, immunostaining of the hypoglossal motor nucleus (HN) suggested that ROCK2 is the main isoform expressed in hypoglossal MNs (HMNs; Figure 1A), whereas ROCK1 is the predominant one in synaptic structures (González-Forero et al., 2012). Based on these premises, we hypothesized that ROCK2/TASK1 signaling is central in the regulatory action of several neuromodulators on MN IME.

Here, we found that ROCK, primarily ROCK2, is essential for accurate shaping of the inspiratory-related discharge of HMNs *in vivo*. *In vitro* approaches indicated that ROCK2 impacts MN IME by regulating TASK1 currents. Finally, this ROCK isoform mediates the impact of LPA, thyrotropin-releasing hormone (TRH), and serotonin (5-HT, 5-hydroxytryptamine) on the background membrane conductance. A model is depicted whereby modulation of TASK1 by ROCK2 is a convergence point in the regulation of intrinsic neuronal excitability by several neurotransmitters and neuromodulators.

## Materials and Methods

The animals used in this study were delivered by the local Animal Supply Services (SEPA, University of Cadiz). The care and handling of animals followed the guidelines of the European Union Council (2010/63/EU, 86/609/UE) on the use of laboratory animals. The experimental procedures were approved by the Ministry of Agriculture, Fisheries, and Rural Development (Junta de Andalucía, Spain). Experiments were performed in neonatal (P6-P9, either sex) and adult Wistar rats. Specifically, neonatal (P6-P9, either sex) mice of C57BL/6J (*wt*), *task1*^–/–^, and *task3*^–/–^ genotypes, and C57BL/6J, *task1*^–/–^, and *task3*^–/–^ pregnant mice (12.5 days gestation). The *task1*^–/–^ and *task3*^–/–^ mice, given by Dr. Douglas A. Bayliss (University of Virginia, VA, United States), became established colonies in SEPA. Animals were individually housed in cages with water and food pellets available *ad libitum*, at 21 ± 1°C, with a 12-h light/dark cycle. Neonatal animals were housed with their mother. Ethical principles were applied to minimize the number of animals used and their suffering. Surgical processes were carried out under aseptic conditions.

### Immunohistochemistry

Sampling and immunohistochemical processing have been performed as described previously with minimal modifications (Portillo and Moreno-López, 2020). Briefly, adult rats were anesthetized with chloral hydrate, injected intraventricularly with heparin, and perfused transcardially, first with PBS, followed by 4% paraformaldehyde in 0.1 M phosphate buffer (PB), pH 7.4, at 4°C. The brains were removed and postfixed for 2 h in the same fixative solution. Brains were cryoprotected by overnight immersion in 30% sucrose in 0.1 M PB at 4°C. Serial coronal sections (30 μm-thick) were obtained from brainstems using a microtome and stored at –20°C in a cryoprotectant solution (glycerol/PBS, 1:1 v/v) until histological processing.

Immunohistochemistry against ROCK1 or ROCK2 was performed as in our previous report (González-Forero et al., 2012). Sections were rinsed in PBS and immersed in 2.5% (w/v) bovine serum albumin, 0.25% (w/v) sodium azide, and 0.1% (v/v) Triton X-100 in PBS for 30 min, followed by overnight incubation at 4°C with each of indicated antisera. Polyclonal primary antibodies used in this study were anti-ROCK1 (1:50; Santa Cruz Biotechnology, Cat# sc-6055, RRID:AB_2182155) and anti-ROCK2 (1:50 or 1:100; Santa Cruz Biotechnology, Cat# sc-1851, RRID:AB_2182311) developed in goat. Subsequently, the tissue was rinsed three times with PBS for 5 min each and incubated for 1 h at room temperature with Cy3-conjugated or biotinylated anti-goat IgGs (1:400; Jackson ImmunoResearch Laboratories) as secondary antibodies. Finally, sections were washed with PBS and mounted on slides with a solution of propyl gallate (0.1 mM in PBS/glycerol, 1:9 v/v) for immunofluorescence analysis. Alternatively, biotin was detected by means of the avidin-biotin-peroxidase system (Pierce, Rockford, IL, United States) using chromogen 3,3-diaminobenzidine tetrahydrochloride. These sections were mounted on slides, dehydrated, covered with DePeX, and visualized under light microscopy. Omission of the primary antibodies resulted in no detectable staining.

Sections were analyzed using an Olympus IX81 inverted microscope for light microscopy or an Olympus FV1000-MPE confocal microscope for fluorescence microscopy (Olympus, Japan). For comparison between different experimental conditions under light microcopy, acquisition setting was kept identical. Animals and tissue were processed in parallel. Images were processed for background subtraction to obtain the maximum dynamic range of grayscale (from 0 to 250) and were analyzed using the software provided by Olympus. In all cases, the area delimiting the HN was manually traced and the mean optical density (o.d.) was measured. HNs were acquired in several sections obtained from at least three animals per treatment.

### Unitary Extracellular Recordings of Hypoglossal MNs in the Adult Rat

Adult rats were prepared for extracellular recordings and microiontophoretic administration of drugs as previously reported (Montero et al., 2008). Three-barreled, microfilament-filled glass pipettes, pulled and broken to a diameter of 5–7 μm, were used for single-unit recording and iontophoresis. Multibarrel glass micropipettes were placed under visual guidance and advanced through the brainstem into the HN. The correct positioning of the micropipette was confirmed by recording the characteristic inspiratory pattern and the presence of the antidromic field potential elicited by electrical stimulation of the ipsilateral XIIth nerve. HMNs were identified by their antidromic activation from the XIIth nerve and by the collision test (González-Forero et al., 2004). The electrical signals were amplified and filtered at a bandwidth of 10 Hz–10 kHz for display and digitization purposes.

The recording barrel (1–3 MΩ) was filled with 3 M NaCl. Depending on the experimental series, remaining barrels were filled with the ROCK inhibitor (*S*)-(+)-2-methyl-1-[(4-methyl-5-isoquinolinyl)sulfonyl]-hexahydro-1*H*-1,4-diazepine dihydrochloride (H1152, 20 mM), (RS)-α-amino-3-hydroxy-5-methyl-4-isoxazolepropionic acid (AMPA; 400 μM), the saturated form of LPA (18:0, sLPA; 1 or 5 mM) or, alternatively, with the vehicle (PBS at pH 8.0) solution. Curves of neuronal responses to iontophoretic current pulses of drugs or vehicle were obtained by applying increasing currents (20–140 nA, 20 nA steps, 30 s duration) through the corresponding barrels using the Neurophore BH-2 system (Harvard Apparatus). In another group of experiments, H1152 (20 mM, +80 nA) or vehicle (+80 nA) were continuously administered for 60 s. In some trials, iontophoretic application of H1152 (+60 nA) and AMPA (–60 nA) were combined always starting with application of one of them, indistinctly, and subsequent co-ejection of the other one. Retaining currents of ± 5 nA were applied between steps to minimize undesired drug flow from the barrel.

Only the inspiratory HMNs discharging at basal conditions (ET_*C*__*O*__2_ = 4.8–5.2%) were considered in this study. Unitary discharge activity and percentages of expired CO_2_ and O_2_ recordings were amplified, filtered, digitized, and stored on computer using the PowerLab/8SP A/D interface (ADInstruments, Castle Hill, Australia) for off-line analysis. The mean firing rate (mFR) (spikes/s, sp/s) averaged over the duration of the inspiratory burst and burst duration were measured. The interval between the beginning of two consecutive bursts was used to calculate the burst rate (burst/min) indicative of breathing rate. Bursts were automatically selected using a macro function and parameters were saved in a data pad for subsequent statistical analysis.

### *In vitro* Whole-Cell Electrophysiological Recordings

Whole-cell patch-clamp recordings were performed in HMNs from coronal brainstem slices (P6–P9 rats/mice) or in primary cultures of embryonic spinal cord MNs (SMNs) (García-Morales et al., 2015, 2019). Succinctly, neonatal animals were decapitated under anesthesia by hypothermia (10–15 min at 4°C). Brainstems were rapidly removed and dissected in artificial cerebrospinal fluid (aCSF) enriched with sucrose at 4°C (in mM: 26 NaHCO_3_, 10 glucose, 3 KCl, 1.25 NaH_2_PO_4_, 2 MgCl_2_, and 218 sucrose), and bubbled with 95% O_2_ and 5% CO_2_. Transverse slices (300–400 μm-thick), obtained using a vibroslicer (NVSL; WPI), were transferred to normal oxygenated aCSF (in mM: 26 NaHCO_3_, 10 glucose, 3 KCl, 1.25 NaH_2_PO_4_, 6 MgCl_2_, 130 NaCl, and 0.5 CaCl_2_) and eventually stabilized at ∼37°C for 1 h. Slices were then transferred to a recording chamber for whole-cell patch-clamp recordings or to an incubation chamber for treatment with different drugs before HN microdissection. SMN cultures were prepared as described below.

Recordings of HMNs and SMNs were performed under constant perfusion (∼3–4 ml/min) with normal oxygenated aCSF at 31°C. Recordings were obtained from MN somata, using a Nikon Eclipse CFI60 microscope equipped with IR-DIC (Tokyo, Japan). Patch electrodes (1.5–3 MΩ resistance) were filled with the following internal solution (in mM): 17.5 KCl, 122.5 K-gluconate, 9 NaCl, 1 MgCl_2_, 10 HEPES, 0.2 EGTA, 3 Mg-ATP, and 0.3 GTP-Tris at pH 7.4, respectively. Recordings were obtained and low-pass Bessel were filtered at 10 kHz with a MultiClamp 700B amplifier. Data were digitized at 20 kHz with a Digidata 1332A analog-to-digital converter and acquired using pCLAMP 9.2 software (Molecular Devices, Foster City, CA). Analysis was only performed for recordings with 5–20 MΩ access resistance. Recordings were discarded if access resistance changed by > 15% during the trial. Series resistance was usually compensated at 65–75%. The pipette offset potential was counterbalanced just before MNs were patched. Recordings were not corrected for liquid junction potential.

In the current-clamp configuration, resting Vm, input resistance (R_N_), and current threshold (I_th_) were measured for the assessment of MN IME. R_N_ was calculated from the current-voltage (I–V) plots obtained by injecting a series of depolarizing and hyperpolarizing current pulses (0.5 s; –0.2 to 0.2 nA). The resulting data points were then fitted with a regression line, and R_N_ was estimated as the slope of the lines. I_th_ was determined as the lowest depolarizing current pulse (5 ms) required to elicit an action potential in 50% of cases.

Voltage-clamp recordings were all carried out in presence of tetrodotoxin (TTX, 1 μM; Tocris Cookson, Bristol, United Kingdom). MNs were initially held near the resting potential (–65 mV), and then voltage-clamp protocols consisting either of depolarizing ramps or of command steps, were applied. Initially, the holding current (I_holding_) required to keep Vm at –65 mV was measured. In the experiments designed to evaluate TASK-like pH-sensitive currents, SMNs were sequentially recorded at varying pH levels (pH 6.2, 7.4, and 8.2). Hydrogen ion concentration was adjusted by adding either HCl or NaOH to aCSF. In addition, responsiveness of MNs at pH 7.2 to TRH (10 μM; Sigma) and 5-HT (5 μM; Sigma) applications were tested. The protocols to obtain I-V relationships consisted either of increasing voltage ramps (2 s duration) from –120 to –40 mV or of voltage steps applied in 5 mV increments between –50 and –120 mV from a baseline holding potential of –65 mV. The slope conductance (G_*s*_) was calculated as the slope of the I-V linear fits as generated by the ramp protocol within the voltage range of –100 to –40 mV. Similarly, input conductance (G_N_) was determined as the slope of the I-V linear fits as obtained from current responses to voltage steps. In this case, the instantaneous component was measured in a time window between the settling of the transient capacitive current and the onset of the time-dependent current (I_h_), which is ∼10 ms after the onset of the step. In some trials, ROCK-dependent currents (H1152-sensitive currents) were obtained by subtracting the I–V relationship obtained in H1152-containing aCSF from that was measured in absence of the drug.

### Quantitative Real-Time Reverse Transcriptase PCR (qRT-PCR)

Total RNA was extracted from micro dissected HNs of adult rats or cultured SMNs (100,000 cells per well) using TRIzol (BioLine, Memphis, TN, United States). To reduce DNA contamination, samples were additionally treated with the RNase-free DNase set according to the manufacturer (Qiagen, Hilden, Germany). The concentration and purity of RNA samples were determined by spectrophotometry at 260 and 280 nm, and 0.5 μg of RNA was used for cDNA synthesis with iScript cDNA synthesis (Bio-Rad, Hercules, CA, United States). *q*RT-PCR was performed using iQ SYBR Green Supermix (Bio-Rad) with the MiniOpticon real-time PCR detection system (Bio-Rad). The PCR primers were as indicated in Supplementary Table 1. In all cases, the validity of amplification was confirmed by the presence of a single peak in the melting temperature analysis and linear amplification with increasing number of PCR cycles. Control samples obtained by omission of RT were used to detect potential contaminations with genomic DNA. Amplification was absent in these RT(-)-controls for all the primers. The cDNA levels for the different samples were determined using the 2^–ΔΔCt^ method, using *gapdh* as the housekeeping gene. All analyses were performed in triplicate, with each experiment repeated at least twice.

### Primary Cultures of Spinal Cord MNs

Primary cultures of SMNs were prepared from the spinal cord of mouse embryos at 12.5 days of gestation (E12.5), following a well-established protocol in our lab (García-Morales et al., 2015, 2019). Isolated cells were pooled in a tube containing culture medium and plated. Cultured SMNs were clearly identified by immunofluorescence using the SMI32 antibody or by morphological criteria. Isolated SMNs were plated either in 4-well tissue culture dishes (Nunc, Thermo Fisher Scientific, Roskilde, Denmark) for *q*RT-PCR experiments (100,000 cells per well) or on 24 mm Corning glass coverslips (Corning, NY, United States) placed in 35 mm culture dishes for electrophysiological recordings (19,000 neurons per well). Culture medium was Neurobasal (Gibco, Invitrogen, Paisley, United Kingdom), supplemented with B27 (Gibco; Invitrogen), horse serum (2% v/v), L-glutamine (0.5 mM), 2-mercaptoethanol (25 μM; Sigma-Aldrich), and a cocktail of recombinant neurotrophic factors: 1 ng/ml brain derived neurotrophic factor, 10 ng/ml glial cell-line derived neurotrophic factor, 10 ng/ml ciliary neurotrophic factor, and 10 ng/ml hepatocyte growth factor (PreProtech, London, United Kingdom). RNA extraction or electrophysiological recordings were performed 6 days after plating (6 DIV). Data were obtained from at least three independent cultures.

### Rho-Associated Coiled-Coil-Containing Kinases Activity Assays

Brainstem slices or SMNs were obtained and incubated as described in this section. The HNs were micro dissected from brainstem slices and immediately immersed in 20 mM Tris pH 8, 250 mM sucrose (70 μl). Samples were then homogenized using an insulin syringe, and subsequently centrifuged at 1,500 x g for 5 min to remove nuclei. Supernatant was used to measure ROCK activity with the 96-well ROCK Activity Assay Kit (Cell Biolabs, San Diego, CA, United States) according to the manufacturer’s instructions. Kit consisted in an enzyme immunoassay developed for the detection of the specific phosphorylation of MYPT1 at Thr^696^ by ROCK. A strip well microtiter plate is pre-coated with a recombinant MYPT1. After incubating the substrate wells with ROCK samples (such as purified kinase, cell, or tissue lysate), the phosphorylated MYPT1 is detected by an anti-phospho-MYPT1 (Thr^696^) antibody. The kit has detection sensitivity limit of 200 pg of active ROCK2. A recombinant active ROCK2 was also provided as a positive control. For this assay, SMNs were allowed to differentiate for 6 days in 6-well tissue culture dish (Cultek), and then they received different drugs for 10 min. In some experiments, SMNs were incubated with different oligonucleotides from 2 DIV (see below). Untreated and treated SMNs were washed with PBS and scraped into the buffer described above. The subsequent procedures were identical to those described for HNs.

### Drugs and Treatments

Oligonucleotides administration to adult rats was as we have previously defined (García-Morales et al., 2015, 2019). Animals were anesthetized (1.5–3.0% isoflurane in 100% O_2_) and placed in a Kopf stereotaxic instrument. The needle of a micro syringe (5 μl, Hamilton Company, Tokyo, Japan) crossed the skull through the middle point of the interparietal-occipital suture and was advanced parallel to occipital bone up to the fourth ventricle. The final position of the needle ending was confirmed visually by means of a surgical microscope. The animals then received a single injection of a small-interfering RNA against *rock1* (siRNA*_*rock*__1_*), *rock2* (siRNA*_*rock*__2_*), or of a non-targeting siRNA (cRNA) (5 μg/rat; Accell, Dharmacon Inc., Lafayette, CO, United States) in 5 μl of RNase-free PBS at a rate of 0.5 μl/min. The target sequences for siRNAs were as detailed in Supplementary Table 1. After the injection, the needle was left in place for 5 min, then was slowly removed. Subsequently, the incision was sutured, cleaned with an aseptic solution (povidone-iodine), and the animals were allowed to survive 3–5 days for *q*RT-PCR, immunohistochemistry, or electrophysiological recording techniques. All animals received one post-operative injection of penicillin (20,000 i.u./kg; i.m.) in order to prevent infection. Pirazolone (0.1 mg/kg; i.m.) was given on awakening for post-operative analgesia.

In SMN cultures, oligonucleotides (cRNA, siRNA*_*rock*__1_*, siRNA*_*rock*__2_*; 2 μM each) were added to the culture medium at 2 DIV and washed out at 5 DIV. RNA extraction for *q*RT-PCR analysis or patch-clamp recordings was performed at 6 DIV as follows.

For patch-clamp recordings, brainstem slices at the level of the HN and SMNs were initially perfused for 10 min with normal aCSF to obtain baseline control data (before condition). Next, slices or cultures were super fused for 10–15 min with aCSF supplemented with H1152 (20 or 100 μM; Tocris), sLPA (40 μM; Avanti Polar Lipids Inc., Alabaster, AL), or both together before voltage or current responses were acquired again. Finally, a last round of acquisition was taken after a 10 min washout with drug-free aCSF. In an experimental series, continuous recordings of I_holding_ at –65 mV (see above) were carried out to initially analyze sensitivity of SMNs to extracellular pH changes, and to subsequently evaluate the effect of H1152 (20 μM) added to the bath during super fusion with the acidic aCSF. Chart recordings of I_holding_ were also performed to analyze responsiveness to (i) TRH (10 μM, Sigma-Aldrich) or 5-HT (5 μM, Sigma-Aldrich) applications in the absence or under the continuous presence of either H1152 or the specific G_αq_ inhibitor YM-254890 (1 μM, a generous gift from Taiho Pharmaceutical Co, Tsukuba, Ibaraki, Japan) in HMNs, and (ii) to the same neuromodulators in siRNA*_*rock*1_*-, siRNA*_*rock*2_*-, or cRNA-treated SMNs. All drug-induced alterations were reversed upon washing, and hence, in most figures, this condition has been omitted for clarity. In some experiments, the human constitutively active ROCK2 (aROCK2; 4.36 μg/ml; 0.069 nM; Sigma-Aldrich) or the specific inhibitor of G_αi/o_ pertussis toxin (PTX; 100 ng/ml; Calbiochem) were present in the internal patch pipette.

### Statistics

Summary data are all presented as mean ± SEM. The number of analyzed specimens per experimental paradigm, and statistical tests applied to each data set are indicated in figure legends or in the results section. Statistical analysis was performed using SigmaPlot (Systat Software, Inc.). The minimum significance level was set at *p* < 0.05. Statistical tests were employed for all data sets with similar variance. For comparison between two groups, normally distributed data were analyzed by unpaired or paired Student’s *t*-test, unless otherwise stated, while non-parametric data sets were assessed by Mann-Whitney *U*-test. One-way or two-way ANOVA followed by *post hoc* Holm-Sidak method were employed for comparison of three or more groups which passed normality test. No data points were excluded from the statistical analysis unless otherwise noted.

## Results

### Rho-Associated Coiled-Coil-Containing Kinase Is Essential to Maintain Discharge Activity Driven by AMPA Receptor Activation in Hypoglossal MNs *in vivo*

As previously reported (González-Forero et al., 2012), immunohistochemistry supported that HMNs in the adult rat mainly express the ROCK2 isoform (Figure 1A), even though residual expression of the alternative homomer ROCK1 in these neurons cannot fully be discarded. In this line, *q*RT-PCR analysis confirmed that*rock2* mRNA was ∼7.5-fold more abundant that *rock1* mRNA in SMNs at 6 DIV (Supplementary Figure 1). On this basis, we first investigated whether ROCK is physiologically necessary for shaping and maintaining basal discharge activity of HMNs *in vivo*. Here, we took advantage that in the decerebrated rat model, the inspiratory-related afferent activity on HMNs persists (Hwang et al., 1983a,b). Therefore, an experimental series was conducted to study the effect of microiontophoretic application of the ROCK inhibitor, H1152, on inspiratory-related activity of antidromically-identified HMNs that were subjected to unitary extracellular recordings at basal conditions (ET_*CO*2_ = 4.8–5.2%). We analyzed the time course of the mean firing rate averaged over the duration of each inspiratory burst (mFR/burst) during application of progressively increasing currents (+ 20 to + 80 nA, 30 s duration) through the drug barrel (Figure 1B, left). A current-dependent decrease in the mFR/burst was observed for H1152 but not when current was applied throughout vehicle solution (Figure 1B, right). Maximal reduction was reached at the highest current applied (–66.2 ± 6.9%; + 80 nA). By using this same current magnitude, HMNs were also tested during sequential application of 60 s duration pulses of ejection current delivered first through the vehicle solution, and then via the H1152-containing barrel. Under this experimental paradigm, H1152 induced a strong reduction (–78.6 ± 5.8%) in mFR/burst in comparison to preceding current injection through vehicle (–9.1 ± 2.4%) solution (Figure 1C).

The inspiratory synaptic drive to HMNs is mediated by the excitatory amino acid glutamate mainly acting on AMPA receptors (AMPARs) (Rekling and Feldman, 1998; Feldman et al., 2005). Therefore, H1152 mainly affects spike discharge activity, which was evoked by AMPAergic neurotransmission on HMNs. To strengthen this idea and to provide support on a feasible postsynaptic role of ROCK, we microiontophoretically applied the analogue AMPA to specifically potentiate AMPA-ergic signaling, while eluding most presynaptic effects. As expected, AMPA application significantly induced both an increase in the mFR/burst (+ 29.5 ± 5.7%) and the emergence of spikes (sp) in the inter-burst interval (mFR: 23.0 ± 4.5 sp/s) (Figures 1D–G). Strikingly, delayed co-ejection of H1152 not only avoided AMPA effects but also reduced mFR/burst to levels comparable to those measured under H1152 alone (–62.2 ± 6.7%). ROCK inhibitor almost entirely occluded the AMPA-induced action potentials generation between inspiratory-related bursts (2.2 ± 1.0 sp/s) (Figures 1D–G). In addition, preceding ejection of H1152 induced a strong reduction in mFR/burst (–79.8 ± 7.2%), which was unaltered by subsequent AMPA co-application (–86.6 ± 3.8%) (Figures 1E,F). Altogether, these outcomes indicate that ROCK is essential for shaping and coding inspiratory-related activity that is driven by excitatory AMPA-ergic signaling neurotransmission in HMNs.

### Interfering With ROCK2 Impacts Inspiratory-Related Activity in Hypoglossal MNs *in vivo*

**FIGURE 2:**
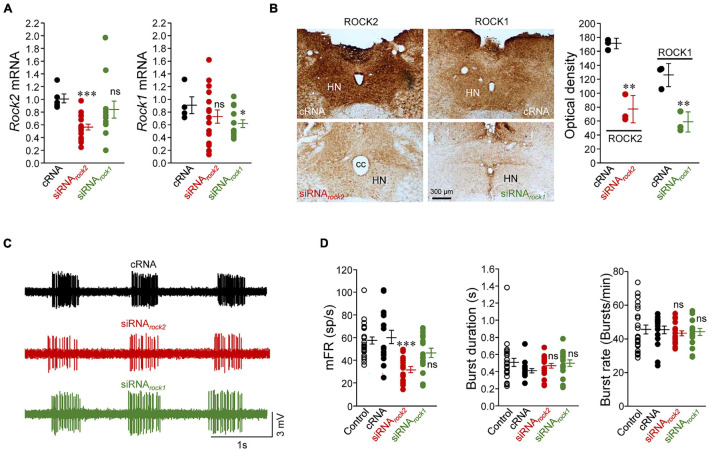
Knockdown of ROCK2, but not ROCK1, led to a reduction in the inspiratory-related discharge activity of HMNs *in vivo*. **(A)** Expression levels of mRNA for the two ROCK isoforms obtained by *q*RT-PCR in micro dissected HNs from adult rats relative to the housekeeping *gapdh*. Tissue extraction was performed 3–5 days after microinjection of the indicated oligonucleotides (5 μg/5 μl) into the fourth ventricle. For *rock2* mRNA: cRNA, *n* = 6 wells; siRNA*_*rock*2_*, *n* = 18 wells; siRNA*_*rock*1_*, *n* = 12 wells. For *rock1* mRNA: cRNA, *n* = 4 wells; siRNA*_*rock*2_*, *n* = 18 wells; siRNA*_*rock*1_*, *n* = 13 wells. *n* ≥ 3 rats per condition. **(B)** Low-magnification bright-field images (left) and optical density (in arbitrary units) of immunolabeling against ROCK2 and ROCK1 in the HN of adult rats previously microinjected (as in **A**) with the indicated oligonucleotides. *n* = 3 rats per condition. **(C)** Extracellularly recorded spike discharge for three HMNs displaying the characteristic inspiratory-related burst of action potentials under the indicated siRNA treatments. Recordings were made from animals at normocapnic conditions (ET_*CO*2_ = 4.8–5.2%). **(D)** Mean values of mFR per burst (left), burst duration (middle), and burst rate (right), indicative for breathing rate, estimated from the recordings under each indicated treatment. Control, *n* = 24 HMNs; cRNA, *n* = 16 HMNs; siRNA*_*rock*2_*, *n* = 18 HMNs; siRNA*_*rock*1_*, *n* = 16 HMNs. Error bars, SEM. ^∗^*p* < 0.05, ^∗∗^*p* < 0.01, ^∗∗∗^*p* < 0.001, ns, not significant; by unpaired Student’s *t*-test **(A)**, non-parametric Mann-Whitney *U*-test **(B)**, or one-way ANOVA with *post hoc* Holm-Sidak method **(D)**.

The role of each one of the two ROCK isoforms in maintaining the respiratory-related discharge in HMNs was further investigated by means of the small-interfering RNA (siRNA) technology. We first confirmed the efficacy and specificity of siRNAs against mRNAs for *rock1* (siRNA*_*rock*1_*) or *rock2* (siRNA*_*rock*2_*) by *q*RT-PCR analysis of mRNA extracted from SMNs (Supplementary Figure 2). Three days after infusion of siRNA*_*rock*2_* into the fourth ventricle, a reduction in*rock2* mRNA (–44.5 ± 4.4%), but not in *rock1* mRNA (–16.8 ± 13.4%), was clearly shown in micro dissected HNs as compared with non-targeting siRNA (cRNA) which was taken as control (Figure 2A). Otherwise, siRNA*_*rock*1_* reduced the corresponding mRNA (–25.1 ± 5.6%) but did not alter significantly *rock2* mRNA (–15.1 ± 8.9%) levels (Figure 2A). As a result, optical density of immunostaining against ROCK1 or ROCK2 in the HN was lower after the siRNA*_*rock*1_* (–55.0 ± 6.6%) or siRNA*_*rock*2_* (–53.4 ± 6.6%) treatment, respectively, than after the cRNA injection (Figure 2B). As expected from the predominant expression of ROCK2 in HMNs, whilst siRNA*_*rock*2_* administration led to a significant depression in mFR/burst (–48.4 ± 4.6%), this parameter was unchanged after siRNA*_*rock*1_* treatment (mFR/burst: –23.7 ± 6.9%), as compared to control condition (Figures 2C,D). Interestingly, neither of the two oligonucleotides tested was found to alter burst duration or burst rate (Figures 2C,D) with the latter revealing that the integrity of premotor structures was similar in all experimental conditions. Therefore, these results strongly supported that ROCK2 is a necessary partner for the precise processing of the incoming AMPAR-mediated, inspiratory-related drive to HMNs.

### aROCK2 Increases Hypoglossal MN Intrinsic Membrane Excitability Depending on TASK1 Subunits

Although the results derived from microiontophoretic application of AMPA, along with those obtained in siRNA*_*rock*2_* treated HMNs (which represent the main *locus* of ROCK2 expression in the HN) will likely reflect a prevalently postsynaptic effect of the inhibition/downregulation of this enzyme isoform, a presynaptic action should not be discarded at all since (i) ROCK activity modulates neurotransmitter release from excitatory glutamatergic presynaptic terminals contacting HMNs (Moreno-López et al., 2011; González-Forero et al., 2012), (ii) ROCK2 could be present also in presynaptic terminals (González-Forero et al., 2012), and (iii) in addition to their classical postsynaptic localization, AMPARs can also be present in presynaptic terminals, where they have been suggested to modulate neurotransmitter release (Schenk and Matteoli, 2004).

**FIGURE 3:**
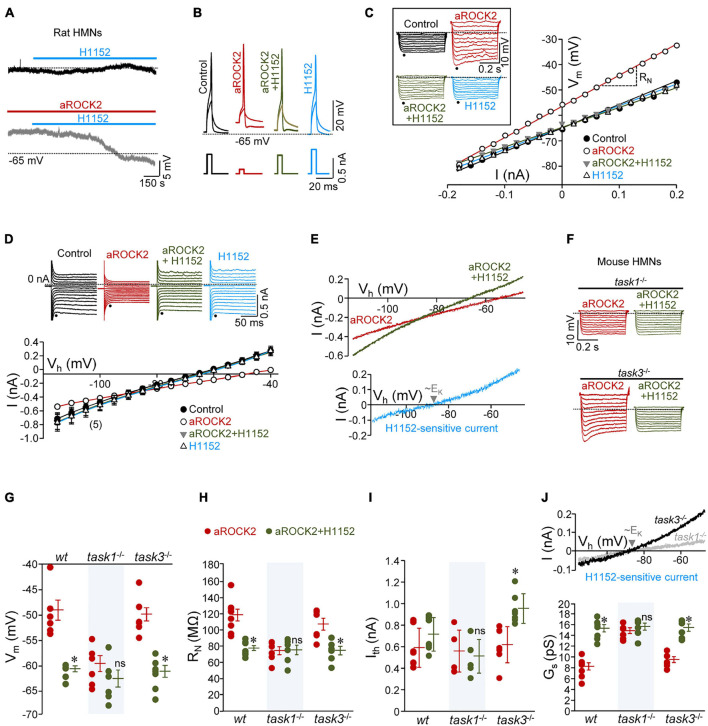
Intracellular constitutively active ROCK2 (aROCK2) increases HMN intrinsic membrane excitability (IME) through inhibition of TASK1, but not TASK3, background K^+^ channels. **(A)** Effect of addition to the bath solution of H1152 (20 μM) on the Vm of two HMNs in slices from P7 rat pups without (top) or with aROCK2 (4.36 μg/ml) into the internal solution of the recording pipette. **(B)** Examples of voltage responses to short (5 ms) depolarizing current pulses (applied at I_th_) obtained in different HMNs under the specified recording conditions. **(C)** V-I relationship for the voltage responses (shown in the inset) to a series of hyperpolarizing current pulses (0.5 s duration, 0.02 nA increments) obtained in the same HMNs as in **(B)**. R_N_ was determined by the slope of the regression line through the V-I plot. Dots in the inset indicate the time point used to measure the peak voltage response. **(D)** Top, examples of current responses to voltage-step commands (range, –120 to –50 mV; 5 mV increments) recorded at pH 7.2 from representative HMNs subjected at indicated conditions. Instantaneous currents were measured at the end of the capacitive transient (dots). Bottom, averaged data (*n* = 5 HMNs per condition) of instantaneous I–V relationships in the control and treated groups. G_N_ values were calculated as the slopes of the linear regression lines fitted to I–V plots. **(E)** Currents evoked by ramping the membrane from –120 to –40 μV (40 mV/s) plotted against holding potential (Vh) obtained from a HMN during recording with aROCK2-containing internal solution before and after addition of H1152 to the bath solution. Linear regression analysis (*r* > 0.9) was used to calculate G_*s*_ from these I–V relationships. Inset, I–V relationship of the H1152-sensitive current obtained by graphical subtraction of ramp currents under each condition. Note that current reversed between near –90 mV, which was close to the reversal potential predicted theoretically by the Nerst equation for K^+^ (E_*K*_). **(F)** As in **(C)** (inset), but for HMNs recorded in slices obtained from *task1*^–/–^ and *task3*^–/–^ knockout mice at P7-P9. **(G–J)** Mean values of Vm (**G**, *wt*, *n* = 7 HMNs; *task1*^–/–^, *n* = 6 HMNs; *task3*^–/–^, *n* = 7 HMNs), R_N_ (**H**, *wt*, *n* = 7 HMNs; *task1*^–/–^, *n* = 6 HMNs; *task3*^–/–^, *n* = 7 HMNs), I_th_ (**I**, *wt*, *n* = 6 HMNs; *task1*^–/–^, *n* = 6 HMNs; *task3*^–/–^, *n* = 7 HMNs), and G_*s*_ (**J**, bottom, *wt*, *n* = 6 HMNs; *task1*^–/–^, *n* = 5 HMNs; *task3*^–/–^, *n* = 6 HMNs) for HMNs from each genotype under the specified pharmacological conditions. Note the lack of effects of H1152 on these parameters in *task1*^–/–^ HMNs. The H1152-sensitive current (**J**, top) was evidenced in *task3*^–/–^ but almost negligible in *task1*^–/–^. Error bars, SEM. ^∗^*p* < 0.05; ns, not significant; by paired Student’s *t*-test.

To better analyze and isolate the possible contribution of postsynaptic ROCK2 in facilitating HMN responsiveness and firing, we performed whole cell patch-clamp recordings of HMNs in *in vitro* brainstem slices from neonatal rats (P6-P9). Given that ROCK inhibits background K^+^ channel TASK1 in human pulmonary artery smooth muscle cells (Seyler et al., 2012) and that this subunit is highly expressed in MNs where it has a profound impact on IME (Talley et al., 2001; Berg et al., 2004; González-Forero et al., 2007), we hypothesized that ROCK2 might also be involved in modulation of HMNs discharge pattern by controlling IME via TASK1. To address this postulate, we first tested the effect of a ROCK inhibitor (H1152, 20 μM) on HMN IME. Recordings revealed, however, that addition of H1152 to the bath solution for 10 min did not alter resting Vm, I_th_ (current threshold to elicit an action potential in 50% of cases), R_N_ (input resistance), G_N_ (slope of the *I–V* curve generated by a voltage-step protocol), or G_*s*_ (slope of the *I–V* curve generated by a voltage-ramp protocol) of recorded HMNs (Figures 3A–E and Supplementary Tables 2, 3). Even though H1152, at this same concentration, has significantly reduced ROCK enzymatic activity in micro dissected HNs (González-Forero et al., 2012), our *in vitro* electrophysiological outcomes support that endogenous baseline ROCK activity, if any in HMNs, was not enough to affect membrane parameters in our experimental conditions.

Then, to investigate the feasible impact of ROCK2 on these membrane properties, we initially opted to add a constitutively active variant of ROCK2 (aROCK2) to the internal solution of the recording micropipette. Once 10 min of stabilization has elapsed after establishing whole cell configuration, recorded HMNs displayed Vm depolarization (+ 7.0 ± 3.6 mV), increased R_N_ (+ 24.5 ± 5.6 MΩ), decreased I_th_ (–0.38 ± 0.07 nA), G_N_ (–3.7 ± 0.6 pS), and G_*s*_ (–3.7 ± 0.7 pS) relative to the control condition (Figures 3A–E and Supplementary Table 2). These data indicate that aROCK2 increases HMN IME by altering background conductance, most probably by closing “leak” K^+^ channels. Strengthening the idea that changes in IME were actually due to aROCK2 activity, addition to the bath solution of H1152 fully reverted to the control state of all analyzed parameters (Figures 3A–E and Supplementary Tables 2, 3). Interestingly, the H1152-sensitive current, generated by subtracting the *I-V* curve obtained during activation of ROCK2 (aROCK2 condition) from that obtained under inhibition of ROCK (aROCK2 + H1152 condition), reversed close to the reversal potential theoretically predicted by the Nerst equation for K^+^ (E_*K*_, approx. –90 mV) in our experimental settings (Figure 3E).

Whether aROCK2 impacts HMN IME by means of TASK1 was addressed by carrying out additional studies in knockout mice for *task1* (*task1*^–/–^). Animals lacking for *task3* (*task3*^–/–^) were taken as a supplementary control to gain in support on the specificity of aROCK2 signaling. Like in rat pups, H1152 did not change Vm, R_N_, I_th_, and G_*s*_ of recorded HMNs from wild-type (*wt*), *task1*^–/–^, or *task3*^–/–^ neonatal mice (P6-P9) (Supplementary Tables 4, 5). Nevertheless, aROCK2 dialyzed from patch pipette into the cell has altered these parameters in *wt* HMNs, with the exception of I_th_ (ΔI_th_: –0.10 ± 0.07 nA), in the same direction and magnitude as in rat HMNs (ΔVm: + 8.3 ± 3.0 mV; ΔR_N_: + 23.5 ± 7.3 MΩ; ΔG_*s*_: –5.6 ± 0.7 pS) (Supplementary Table 4). Conclusively, aROCK2-induced alterations persisted in *task3*^–/–^ (ΔVm: + 11.7 ± 1.3 mV; ΔR_N_: + 16.7 ± 4.9 MΩ; ΔI_th_: –0.16 ± 0.06 nA; ΔG_*s*_: –2.0 ± 1.1 pS) but were absent in *task1*^–/–^ (ΔVm: + 2.7 ± 1.7 mV; ΔR_N_: –1.8 ± 5.9 MΩ; ΔI_th_: –0.18 ± 0.08 nA; ΔG_*s*_: + 1.1 ± 0.5 pS) HMNs (Supplementary Table 4). H1152 returned these parameters to their corresponding control values in *wt* and *task3*^–/–^ HMNs, indicating that these alterations were attributable to aROCK2 (Figures 3F–J and Supplementary Tables 4, 5). The H1152-sensitive current, obtained as above, was larger in *task3*^–/–^ than in *task1*^–/–^ HMNs in which was almost negligible. In both cases, the reversal potential was also near E_*K*_ (Figure 3J, top).

### Intracellular aROCK2 Promotes TASK1 Inhibition in Spinal Cord MNs

**FIGURE 4:**
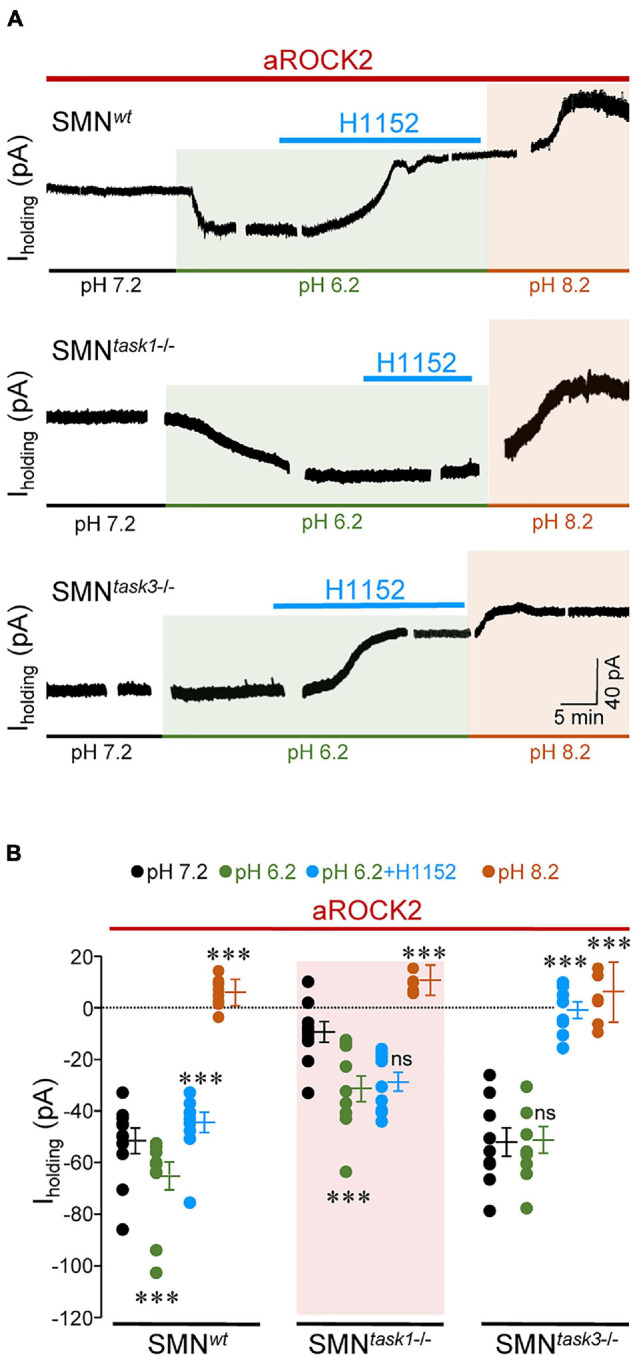
aROCK2 inhibits TASK1 pH-sensitive conductance. **(A)** Time series illustrating changes in I_holding_ for three representative mouse HMNs with indicated genotypes voltage clamped at –65 mV in response to extracellular pH variations and H1152 (20 mM) application. All recordings were performed with aROCK2 in the pipette solution. H1152 was only added to the acidic solution when TASK channels are expected to be almost fully closed. **(B)** Steady-state values of I_holding_ reached after changing the external pH or after adding H1152 to the acidified external solution for spinal cord MNs (SMNs) of each genotype. SMN*^*wt*^*, *n* = 11 HMNs per condition; SMN*^*task*1^*^–/–^: pH 7.2, *n* = 10 HMNs; pH 6.2, *n* = 10 HMNs; pH 6.2 + H1152, *n* = 9 HMNs; pH 8.2, *n* = 8 HMNs; SMN*^*task*3^*^–/–^: pH 7.2, *n* = 9 HMNs; pH 6.2, *n* = 9 HMNs; pH 6.2 + H1152, *n* = 8 HMNs; pH 8.2, *n* = 7 HMNs. Error bars, SEM. ^∗∗∗^*p* < 0.001; ns, not significant; by one-way repeated measures ANOVA with *post hoc* Holm-Sidak method relative to pH 7.2 except for pH 6.2 plus H1152 which was compared with pH 6.2 alone.

To strengthen the idea that aROCK2 affects TASK1-mediated currents, further experiments were performed by using the cell culture model of SMNs. This model was chosen since these cells have an embryonic origin different from HMNs and moreover, its use in *in vitro* patch-clamp studies makes it more feasible to get steady-state recordings for long-lasting experiments. Trials were addressed by modifying extracellular pH to measure TASK-dependent changes in the holding current (I_holding_) required to keep Vm at –65 mV (Duprat et al., 1997; González-Forero et al., 2007). Alkalinization of extracellular medium (pH 8.2) from a physiological pH (7.4) opens TASK channels, enhancing K^+^ efflux, which leads to an outward shift in I_holding_, whereas an acidic solution (pH 6.2) retains K^+^ into the cell by closing TASK channels, which causes an inwardly directed change in I_holding_. Therefore, it is recognized that the impact of pH on I_holding_ depends on the functional state of TASK channels. Voltage-clamp recordings from SMNs at extracellular pH 7.4 with aROCK2 into the pipette solution showed that I_holding_ had higher magnitude in SMNs isolated from *wt* (SMN*^*wt*^*: –51.7 ± 4.7 pA) and *task3*^–/–^ (SMN*^*task*3^*^–/–^: –52.2 ± 5.9 pA) than in those obtained from *task1*^–/–^ (SMN*^*task*1^*^–/–^: –9.5 ± 3.9 pA) embryos (Figure 4). Acidification (pH 6.2) of the extracellular medium led to an inwardly directed shift of I_holding_ in SMN*^*wt*^* (–13.7 ± 5.2 pA), being even emphasized in SMN*^*task*1^*^–/–^ (–21.9 ± 5.4 pA), but absent in SMN*^*task*3^*^–/–^ (+ 0.73 ± 5.6 pA) (Figure 4). Given that MNs mainly express TASK1/3 heterodimers, along with TASK1/1 and TASK3/3 homodimers (Talley et al., 2001; Berg et al., 2004), it would be expected that MNs from knockout mice were enriched in the reciprocal homodimers. Thus, these outcomes strongly support that aROCK2 mainly control the functional state of TASK1 channels promoting inhibition of TASK1-mediated conductance. Reinforcing this notion, addition of H1152 to the acidic solution induced an outward shift of I_holding_ in SMN*^*wt*^* (+ 20.8 ± 3.4 pA) that was accentuated in SMN*^*task*3^*^–/–^ (+ 40.4 ± 3.2 pA) but absent in SMN*^*task*1^*^–/–^ (+ 2.4 ± 3.7 pA) (Figure 4). In a genotype-independent manner, alkalinization of extracellular medium (pH 8.2) brought I_holding_ to a mean value that was similar for the 3 SMN genotypes (SMN*^*wt*^*: 6.4 ± 1.5 pA; SMN*^*task*1^*^–/–^: 10.2 ± 2.1 pA; SMN*^*task*3^*^–/–^: 5.6 ± 4.4 pA) (Figure 4). This last interesting result indicates that mechanisms engaged in TASK1 channel opening under extracellular alkalinization prevails over that mediating aROCK2-induced TASK1 inhibition.

### Lysophospholipid sLPA Increases Hypoglossal MN Intrinsic Membrane Excitability via G_αi/o_/ROCK/TASK1 Signaling

**FIGURE 5:**
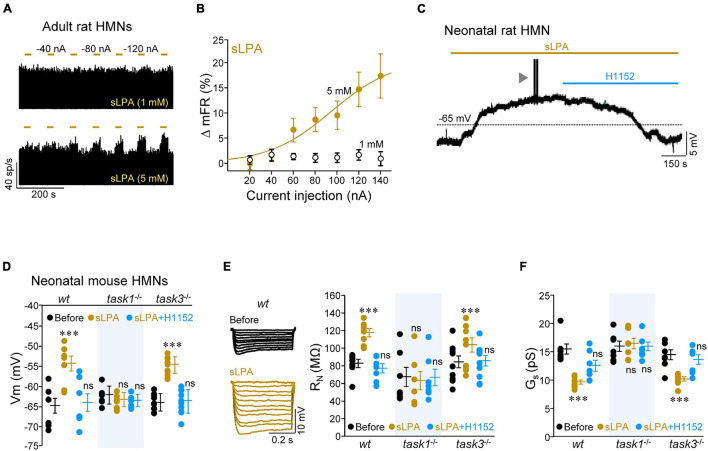
Lysophosphatidic acid (sLPA) increases HMN IME via ROCK/TASK1 signaling. **(A)** Time course of the mFR (sp/s) per burst for two HMNs during microiontophoretic administration (30 s on, 60 s off) of sLPA (1 mM, top; 5 mM, bottom) at the indicated applied currents. **(B)** Mean current-response curves illustrating the effects of the two concentrations of microiontophoretically-administered sLPA on the mFR per burst of recorded HMNs (1 mM, *n* = 12; 5 mM, *n* = 20). **(C)** Effect of the addition to the bath solution of sLPA (40 μM) and subsequent co-addition of H1152 (20 μM) on the Vm of a HMN in a slice obtained from a P7 rat. Arrowhead signals a burst of action potentials elicited during sLPA-induced depolarization. **(D–F)** Mean values of Vm (**D**, *wt*, *n* = 7 HMNs; *task1*^–/–^, *n* = 7 HMNs; *task3*^–/–^, *n* = 10 HMNs), R_N_ **(E)**, *wt*, *n* = 7 HMNs; *task1*^–/–^, *n* = 7 HMNs; *task3*^–/–^, *n* = 10 HMNs, and G_*s*_ (**F**, *wt*, *n* = 6 HMNs; *task1*^–/–^, *n* = 7 HMNs; *task3*^–/–^, *n* = 7 HMNs) for neonatal (P7-P9) mouse HMNs with the indicated genotypes and receiving stated treatments. Voltage responses to a series of hyperpolarizing current pulses (0.5 s duration, 0.02 nA increments) obtained in a *wt* HMN before and after application of sLPA to the bath solution are illustrated in **(E)** (left). Note that sLPA affected these parameters, in a H1152-sensitive manner, only in *wt* and *task3*^–/–^, but not in *task1*^–/–^, HMNs. Error bars, SEM. ^∗∗∗^*p* < 0.001; ns, not significant; by one-way repeated measures ANOVA with *post hoc* Holm-Sidak method relative to the before conditions.

For the next step in this study, we looked for evidence of whether ROCK mediates the action of some known physiological regulator of MN IME that targets TASK1. In this framework, one of them is the bioactive phospholipid LPA, whose downstream signaling cascades include ROCK stimulation, among others (Choi and Chun, 2013), and regulates MN IME in a TASK1-dependent manner (Gento-Caro et al., 2021a,b). Furthermore, LPA-RhoA/ROCK signaling regulates GABA_*A*_ receptor subunits composition in MNs (García-Morales et al., 2015), supporting that LPA signaling can increase ROCK activity in this cell type. In the next set of experiments, we used a saturated form of LPA (18:0, or sLPA hereinafter), one of the major species in the brain (Sugiura et al., 1999). First, we evaluated the effects of microiontophoretically applied sLPA on the discharge activity of HMNs in the *in vivo* model of decerebrated rats by using two different doses of the drug (1 or 5 mM) administered at progressively increasing ejection currents (–20 to –140 nA, 30 s duration; Figure 5A). In contrast to the effect of the ROCK inhibitor, a current-dependent increase in the mFR/burst of HMNs was observed when sLPA was applied from the 5 mM solution, but not when ejected from the barrel filled with the lower concentration (Figures 5A,B). Maximal increase was reached at the highest current intensity applied (+ 17.4 ± 4.4%; –140 nA).

The effects of sLPA were also tested in the *in vitro* model of brainstem slices from neonatal rats. Addition to the bath solution of sLPA (40 μM) induced a pronounced increase in IME of HMNs from rat pups, characterized by Vm depolarization (+ 11.6 ± 2.4 mV; Figure 5C), R_N_ increase (+ 32.5 ± 9.8 MΩ), and I_th_ reduction (–0.38 ± 0.06 nA) (Supplementary Table 6), just like we recently reported for the monounsaturated species LPA (18:1) (Gento-Caro et al., 2021a). Co-addition to the bath solution of H1152 fully reverted changes in Vm and R_N_, but not in I_th_, to control pre-treatment conditions (Figure 5C and Supplementary Table 6). These results confirm that sLPA-induced effects on HMN IME is, at least in part, mediated by ROCK. LPA affects HMN IME by the LPA receptor 1 (LPA_1_) and LPA_1_-mediated modulation of synaptic neurotransmission in MNs involves G_αi/o_ protein (García-Morales et al., 2015; Gento-Caro et al., 2021a,b). Therefore, we next inspected whether action of sLPA on HMN IME also relies on this G protein. For that purpose, we performed electrophysiological recording experiments by using an internal pipette solution containing the G_αi/o_ inhibitor pertussis toxin (PTX) (100 ng/ml). Under intracellular dialysis with PTX, sLPA did not induce any alteration in the analyzed membrane parameters (Supplementary Figure 3 and Supplementary Table 6), supporting that sLPA impacts HMN IME by binding to a G_αi/o_-protein-coupled receptor, most likely LPA_1_.

Given that we previously showed that LPA impacts MN IME via LPA_1_-TASK1 signaling (Gento-Caro et al., 2021a,b), we performed experiments in knockout animals to determine whether ROCK is a mediator in this signaling pathway. As expected, sLPA led to Vm depolarization (+ 10.5 ± 1.9 mV), increase of R_N_ (+ 34.9 ± 4.9 MΩ), and reduction of I_th_ (–0.54 ± 0.29 nA) and G_*s*_ (–5.8 ± 0.4 pS) in HMNs from *wt* and *task3*^–/–^, but not from *task1*^–/–^ neonatal mice (Figures 5D–F and Supplementary Table 7). Co-addition of H1152 reversed the sLPA-induced effects on HMNs IME from *wt* and *task3*^–/–^ animals, except I_th_ in *wt* HMNs (Figures 5D–F and Supplementary Table 7). Altogether, these results indicate that sLPA alters HMN IME by a mechanism involving G_αi/o_/ROCK/TASK1 pathway.

### ROCK2, but Not ROCK1, Mediates Regulation of Spinal Cord MN Intrinsic Membrane Excitability by sLPA

**FIGURE 6:**
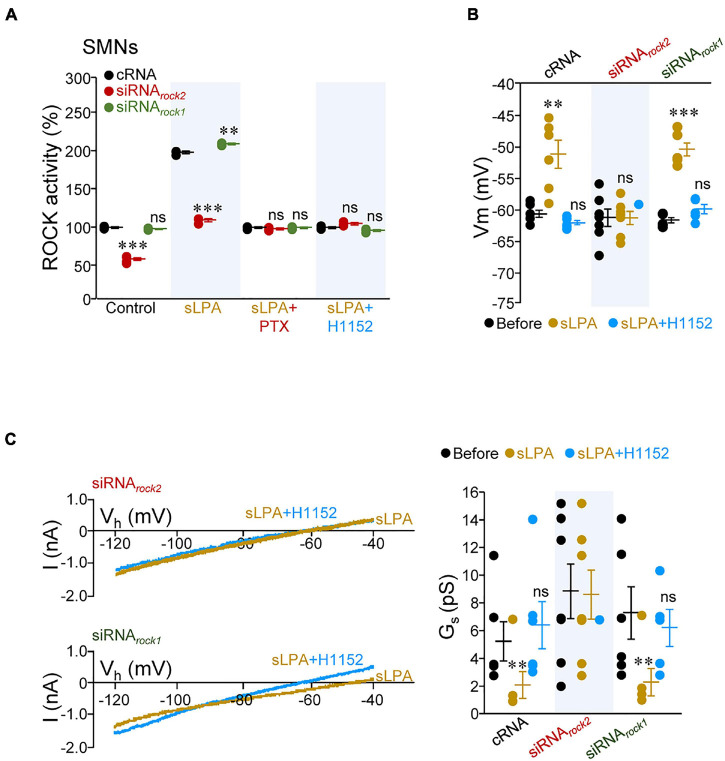
sLPA-induced increase of SMN IME entails G_αi/o_/ROCK2 signaling. **(A)** ROCK activity in homogenates from SMNs at 6 DIV incubated from 2 to 5 DIV with indicated oligonucleotides (2 μM each), untreated (control) or treated with either sLPA (40 μM), sLPA plus PTX (100 ng/ml), or s-LPA plus H1152 (100 mM). All data were relativized taken the control condition of cRNA-treated SMNs as 100%. *n* = 3 assays per condition except for Control/siRNA*_*rock*2_* (*n* = 6 assays). **(B,C)** Mean values of Vm **(B)** and G_*s*_ (**C**, right) obtained from SMNs at 6 DIV pre-treated (as above) with stated oligonucleotides receiving indicated pharmacological treatments. In **(C)** (left), currents evoked by ramping the membrane potential from –120 to –40 mV (40 mV/s) plotted against holding potential (Vh) in two SMNs pre-treated with siRNA*_*rock*2_* (top) or siRNA*_*rock*1_* (bottom) under the presence in the bath solution of sLPA (40 μM) or subsequent sLPA plus H1152 (20 μM) are displayed. cRNA, *n* = 6 SMNs; siRNA*_*rock*2_*, *n* = 7 SMNs; siRNA*_*rock*1_*, *n* = 5 SMNs. Error bars, SEM. ^∗∗^*p* < 0.01, ^∗∗∗^*p* < 0.001; ns, not significant; by one-way ANOVA relative to the cRNA conditions **(A)** or one-way repeated measures ANOVA relative to before conditions **(B,C)** both with *post hoc* Holm-Sidak method.

Identifying the specific ROCK isoform involved in mediating sLPA-induced effects on MN excitability was next addressed. To reach this aim, SMNs were pre-treated with either cRNA, siRNA*_*rock*1_*, or siRNA*_*rock2*_*. In an initial series of experiments, we observed that neither siRNA*_*rock*1_* nor siRNA*_*rock*2_* affected Vm, R_N_, I_th_, or G_*s*_ of SMNs as compared with cRNA treatment (Supplementary Figure 4 and Supplementary Table 8) in spite that pre-incubation of SMN cultures with siRNA*_*rock*2_*, but not with siRNA*_*rock*1_*, led to a reduction in baseline ROCK activity (siRNA*_*rock*2_*: –41.4 ± 1.6%; siRNA*_*rock*1_*: –2.0 ± 0.7%), relative to that measured in cell lysates from cRNA pre-treated SMNs (Figure 6A and Supplementary Table 9). As in brainstem slices, these data indicate that baseline ROCK activity in SMNs is not enough to impact on SMN IME in our experimental conditions. Strikingly, incubation with sLPA increased ROCK activity in cRNA- (+ 98.0 ± 1.6%) and siRNA*_*rock*1_*-treated (+ 109.2 ± 1.0%) SMNs, but not in siRNA*_*rock*2_*-pre-incubated cells (+ 9.5 ± 2.1%). The sLPA-induced stimulation of ROCK in cRNA and siRNA*_*rock*1_* pre-treated groups was prevented by co-incubation with either PTX or H1152 (Figure 6A and Supplementary Table 9). Thus, sLPA signaling in SMNs involves downstream stimulation of ROCK2, but not ROCK1, by acting on a G_αi/o_-protein coupled receptor, most probably LPA_1_ (García-Morales et al., 2015; Gento-Caro et al., 2021a,b).

Accordingly, acute addition of sLPA to the bath solution altered SMN IME in the expected direction in cultures pre-treated with cRNA (Vm: + 9.3 ± 2.2 mV; I_th_: –0.37 ± 0.03 nA; G_*s*_: –3.1 ± 1.0 pS) or siRNA*_*rock*1_* (Vm: + 11.1 ± 1.0 mV; I_th_: –0.17 ± 0.09 nA; G_*s*_: –4.9 ± 1.0 pS) but had no effects in siRNA*_*rock*2_*-treated SMNs (Vm: + 0.2 ± 1.0 mV; I_th_: + 0.01 ± 0.09 nA; G_*s*_: –0.3 ± 1.8 pS). The sLPA-induced alterations in membrane parameters were all reversed by co-addition of H1152, except for I_th_, in the siRNA*_*rock*1_*-treated pool (Figures 6B,C and Supplementary Table 8). Thus, ROCK2 seems the most probable isoform mediating the regulatory action of sLPA on MN IME.

### Several Neurotransmitters Regulate Background Resting Currents Through ROCK2

**FIGURE 7:**
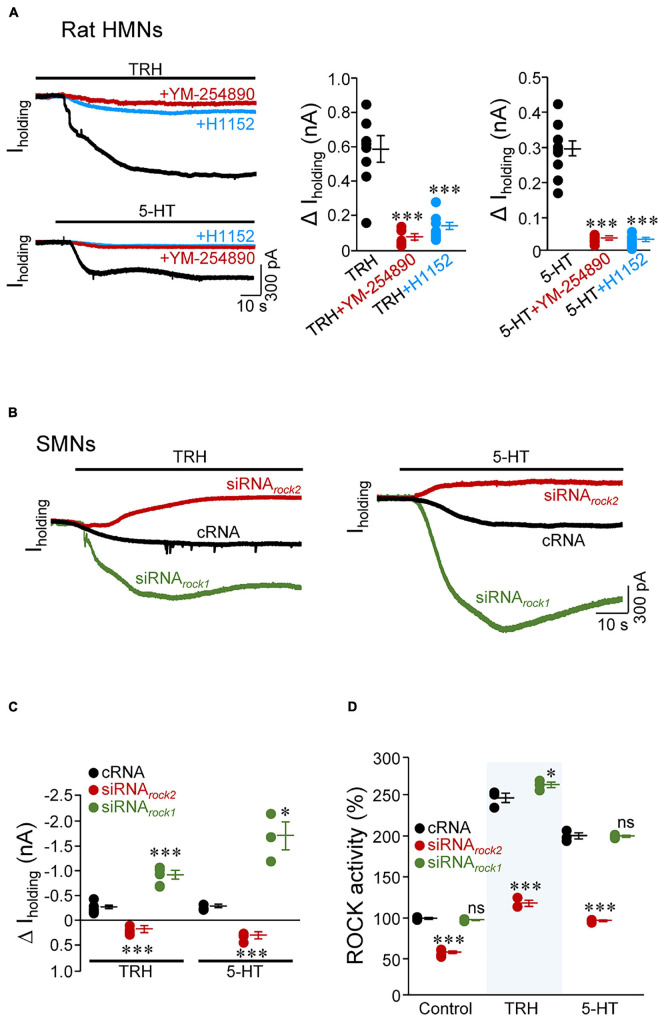
Two neurotransmitters, thyrotrophin-releasing hormone (TRH) and serotonin (5-HT), inhibit background conductance in MNs by activating ROCK2. **(A)** Whole-cell current responses evoked by the application of TRH (10 μM, top) or 5-HT (5 μM, bottom) in representative rat HMNs (P7-P9) in absence (black trace) or under the presence in the bath solution of the G_αq_ inhibitor YM-254890 (1 μM, red trace) or the ROCK inhibitor H11552 (20 μM, blue trace). Membrane potential was held at –65 mV. Maximal quantitative effects are presented in the plots on the right. TRH, *n* = 8 HMNs; TRH + YM-254890, *n* = 7 HMNs; TRH + H1152, *n* = 8 HMNs; 5-HT, *n* = 11 HMNs; 5-HT + YM-254890, *n* = 9 HMNs; 5-HT + H1152, *n* = 8 HMNs **(B,C)** As in **(A)**, but in SMNs at 6 DIV subjected to indicated treatments as in Figure 6A. Quantitative effects are graphically illustrated in **(C)**. For TRH: cRNA, *n* = 5 SMNs; siRNA*_*rock*2_*, *n* = 4 SMNs; siRNA*_*rock*1_*, *n* = 5 SMNs; for 5-HT: cRNA, *n* = 4 SMNs; siRNA*_*rock*2_*, *n* = 4 SMNs; siRNA*_*rock*1_*, *n* = 3 SMNs. **(D)** ROCK activity in homogenates from SMNs at 6 DIV incubated from 2 to 5 DIV with indicated oligonucleotides (2 μM each), untreated (control) or treated with either TRH (10 μM) or 5-HT (5 μM). All data were relativized taken the control condition of cRNA-treated SMNs as 100%. Control condition is the same that in Figure 6A and is presented here for comparison. *n* = 3 assays per condition except for Control/siRNA*_*rock*2_* (*n* = 6 assays). Error bars, SEM. ^∗^*p* < 0.05, ^∗∗∗^*p* < 0.001; ns, not significant; by one-way ANOVA with *post hoc* Holm-Sidak method relative to TRH, 5-HT **(A)**, or cRNA **(C,D)** conditions.

Full inhibition of TASK1 by multiple neurotransmitters, such as 5-HT, norepinephrine, substance P, and TRH, induces slow excitation in MNs (Talley et al., 2000). The proposed mechanism involves channel modulation by G_αq_ through direct interaction with the ion channel or with a closely associated intermediary (Chen et al., 2006). In the voltage-clamp configuration (holding potential: –65 mV), TRH (10 μM) or 5-HT (5 μM) induced an inward shift of I_holding_ in all HMNs tested from rat pup slices (TRH: –575.3 ± 74.0 pA; 5-HT: –296.8 ± 21.0 pA). Addition to the bath of the G_αq_ inhibitor YM-254890 (1 μM) or H1152 strongly reduced magnitude of inwardly directed shift in I_holding_ induced by TRH (YM-254890: –87.8 ± 2.9%; H1152: –76.3 ± 4.6%) and 5-HT (YM-254890: –86.3 ± 1.2%; H1152: –87.8 ± 1.7%) (Figure 7A and Supplementary Table 10). Strikingly, incubation with neurotransmitters led to a strong increase in ROCK activity (TRH: + 153.5 ± 0.2%; 5-HT: + 160.1 ± 3.3%) in homogenates of micro dissected HNs from neonatal rats (Supplementary Table 11). YM-254890 reduced *per se* baseline ROCK activity in the HN (–33.8 ± 0.5%) and prevented neurotransmitters-induced stimulation of ROCK (Supplementary Table 11). These data suggest that G_αq_ could sustain baseline ROCK activity in the HN in our experimental conditions and mediate activation of ROCK downstream to the tested neurotransmitters.

Further, we scrutinized the role of each of the two ROCK isoforms in mediating neurotransmitters impact on I_holding_ in SMNs pre-incubated with different oligonucleotides. TRH (–282.4 ± 50.6 pA) and 5-HT (–278.5 ± 24.0 pA) evoked the expected inwardly directed change of I_holding_ after cRNA treatment. However, in siRNA*_*rock*2_*-treated SMNs, application of any of these neurotransmitters unexpectedly led to a net outward shift in I_holding_ (TRH: + 177.9 ± 39.4 pA; 5-HT: + 301.5 ± 39.7 pA). On the other hand, expected effect of TRH (–911.3 ± 80.2 pA) and 5-HT (–1707.6 ± 276.5 pA) on I_holding_ was markedly accentuated after siRNA*_*rock*1_* administration (Figures 7B,C). Analysis of ROCK activity in SMNs not exposed to TRH and 5-HT revealed that baseline enzymatic activity was affected (–41.4 ± 1.6%) only in the siRNA*_*rock*2_* pre-treatment condition as compared to the cRNA control group. Interestingly, TRH and 5-HT-induced stimulation of ROCK were significantly reduced by siRNA*_*rock*2_* (TRH: –52.0 ± 1.5%; 5-HT: –51.5 ± 0.5%), but remained unaltered after siRNA*_*rock*1_* (TRH: + 6.3 ± 1.4%; 5-HT: –0.1 ± 0.6%), compared to cRNA (Figure 7D and Supplementary Table 12). Altogether, these outcomes are compatible with the hypothesis that both neurotransmitters affect membrane conductance by a mechanism that involves, at least, ROCK2 stimulation. However, the involvement of other kinases in this regulatory pathway, including ROCK1, cannot be discarded (see section “Discussion”).

## Discussion

**FIGURE 8:**
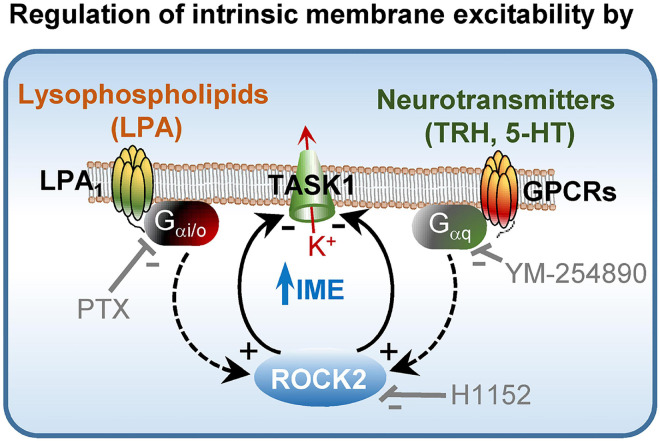
Role of ROCK2 in the control of neuronal IME. Schematic modeling molecular mechanisms by which lysophospholipids and neurotransmitters such as TRH and 5-HT regulate MN IME. Used inhibitors are also indicated in the model. For more details see the text.

From this study, the ROCK2 homolog emerges as a critical partner for accurate handling of afferent drive in MNs. ROCK can contribute to signal processing by regulating neurotransmitter release at the presynaptic counterpart (Moreno-López et al., 2011; González-Forero et al., 2012). Here, we provide solid evidence that ROCK, especially the ROCK2 isotype, also operates at the postsynaptic compartment as an essential mediator in setting up MN IME by modulation of “leak” K^+^ TASK1 channels. In this framework, ROCK2 is an intracellular convergence point in the regulation of MN IME by LPA and several neurotransmitters. These neuromodulators, acting through GPCRs, manage MN IME by mainly regulating TASK1-mediated conductance (Talley et al., 2000; Figure 8). Given the widespread expression of ROCK2 and TASK1 throughout the nervous system (Hashimoto et al., 1999; Talley et al., 2001), this work recognizes a novel intracellular signaling pathway in the fine-tuning of neuronal and network excitability.

Rho-associated coiled-coil-containing kinase (ROCK) is a ubiquitously expressed kinase with two known isoforms. Whilst the ROCK1 homolog is preferentially expressed in kidney, liver, spleen, and testis, ROCK2 is mainly enriched in brain, heart, lung, and skeletal muscle (Hartmann et al., 2015; Liu et al., 2018; Seccia et al., 2020). ROCK2 in bovine brain is profuse in neurons of hippocampus, cerebral cortex, and cerebellum (Hashimoto et al., 1999). *Rock2* mRNA is widely distributed all along the adult mouse central nervous system with high expression levels in HMNs.^1^ In addition, ROCK2 was the main expressed isoform in HMNs and SMNs, whereas ROCK1 was mainly present at presynaptic structures in the HN of the adult rat (González-Forero et al., 2012) and only residual expression of this isoform was detected in SMNs. So far, major functions ascribed to ROCK comprises regulation of several cellular processes such as cytoskeletal organization, cell adhesion and motility, proliferation and apoptosis, remodeling of the extracellular matrix, and smooth muscle contraction (Hartmann et al., 2015; Liu et al., 2018; Seccia et al., 2020). In this context, we have previously reported that neurotransmitter release in the HN is managed by ROCK acting on presynaptic actomyosin apparatus (González-Forero et al., 2012). There is also accumulating evidence that ROCK can negatively or positively regulate different types of ion channels, including TASK1 potassium channels, whose activity is inhibited by direct phosphorylation of the channel by ROCK in smooth muscle cells (Seyler et al., 2012), and, as shown here, by a yet not fully described mechanism in MNs. Based on these findings and on the fact that TASK1 is highly expressed in MNs and has a profound impact on MN IME (Talley et al., 2001; Berg et al., 2004; González-Forero et al., 2007), it can be tentatively proposed that ROCK activity might physiologically determine accurate processing of afferent drive to MNs modulating both presynaptic release of neurotransmitter and postsynaptic responsiveness. In agreement with this idea, microiontophoretic ejection of H1152 drastically reduced inspiratory-related discharge activity of HMNs *in vivo*, which is mostly driven by AMPA-ergic signaling (Rekling and Feldman, 1998). This ROCK inhibitor-evoked depression can be due in part to the reduced glutamate release from synaptic terminals concomitant with the decrement in the size of the readily releasable pool of synaptic vesicles caused by the drug (González-Forero et al., 2012). Concurrently, the finding that H1152 fully occluded action potentials evoked by microiontophoretic application of AMPA, which is expected to be mostly lacking presynaptic effects in the HN, strongly supports an additional postsynaptic action of ROCK in the processing of inspiratory-related afferent drive to HMNs *in vivo*. Particularly, ROCK2 seems to be the isoform involved in this task since interfering with *rock2* mRNA, but not with *rock1* mRNA, led to a depression of breathing-related activity in HMNs. However, it cannot be fully discarded that ROCK1 was also involved in some other aspect related to the processing of afferent information and to the shaping of the discharge activity.

In a precedent report, we had already described that ROCK modulates neurotransmitter release from glutamatergic terminals by a presynaptic mechanism in the HN (González-Forero et al., 2012). Hence, our aim in this study was to address whether ROCK signaling is also modulating intrinsic excitability and responsiveness at the postsynaptic counterpart. Given that ROCK can modulate diverse ionic channels in different tissues (Li et al., 2002; Piccoli et al., 2004; Staruschenko et al., 2004; Iftinca et al., 2007; Seyler et al., 2012; García-Morales et al., 2019), among them TASK1, a feasible action of ROCK on the intrinsic membrane properties of HMNs might also exert a significant influence on the discharge pattern of these MNs. Thus, experiments were performed in slices obtained from rat or mouse pups. Contrary to expected, the results from our *in vitro* slice experiments revealed that baseline ROCK activity was not enough to maintain HMN IME in such experimental conditions, even though it was sufficient to sustain neurotransmitter release (González-Forero et al., 2012). Strikingly, glutamate, throughout binding to AMPAR, but not NMDA receptors, activates RhoA/ROCK signaling in rat hippocampal neuronal cells (Jeon et al., 2002; Kim et al., 2004). Thus, it is probable that in our *in vitro* preparation, spontaneous release of neurotransmitter was not enough to activate ROCK at levels and/or at subcellular locations required to clearly impact on HMN IME. Contrary to this situation, however, during AMPAR-mediated inspiratory-related activity in the *in vivo* preparation, stimulation of RhoA/ROCK could be emphasized downstream AMPARs activation. Furthermore, baseline LPA and several neurotransmitters, which we demonstrate here to increase ROCK2 activity in MNs, could also stimulate ROCK in HMNs in the *in vivo* model.

In an alternative approach, including aROCK2 in the intracellular recording solution resulted in an abrupt increase in HMN IME. Conclusively, aROCK2-induced alterations in IME were absent in HMNs recorded from *task1*^–/–^ but persisted in *task3*^–/–^ mice. In addition, closing TASK channels by acidification of the extracellular medium (González-Forero et al., 2007) in presence of intracellular aROCK2 induced a inwardly directed shift of I_holding_ in cultures from *wt* and *task1*^–/–^ SMNs but had only negligible effects on *task3*^–/–^ SMNs. Given that background conductance in MNs is defined by TASK1/3 heterodimers and by TASK1/1 and TASK3/3 homodimers (Talley et al., 2001; Berg et al., 2004), it would be expected that surface expression of homodimers of the unaffected subunit prevails in knockout animals. This might underlie the lack of phenotypical basic sensorimotor deficits, obvious neurological abnormalities (Aller et al., 2005; Linden et al., 2006; Brickley et al., 2007; Mulkey et al., 2007) or similarities in the intrinsic membrane properties of MNs from TASK knockout mice relative to wild-type mice (Supplementary Table 4). Thus, these outcomes indicate that aROCK2 intracellularly inhibits TASK1-mediated currents. The opening effect of low extracellular proton concentration on TASK1 overcomes intracellular inhibitory action of aROCK2 since, under alkaline pH, the magnitude of outward shift in I_holding_ was similar in SMNs independently of the genotype. Finally, the aROCK-mediated effects on IME of MNs were fully reversed over the course of 10–15 min by bath application of the ROCK inhibitor H1152 and, thus, demonstrating that intrinsic hyper-excitability depended on ROCK activity. The H1152-sensitive current displayed a reversal potential close to theoretical E_*K*_. These results are compatible with a fast regulatory mechanism leading to TASK1 inhibition by aROCK2, such as phosphorylation (Seyler et al., 2012), rather than impairment of TASK1 expression and/or traffic to the plasma membrane which is known to proceed slower (García-Morales et al., 2019). However, further research is needed to clarify the precise mechanism by which aROCK2 inhibits TASK1 currents in MNs.

Lysophosphatidic acid (LPA) is a physiological activator of ROCK by acting on GPCRs (LPARs) (Choi and Chun, 2013). In our previously published paper, we demonstrated that microiontophoretic application of several LPARs inhibitors affected inspiratory-related activity of HMNs *in vivo* (García-Morales et al., 2015). In this study, delivery of sLPA, by using the same method, induced a current-dependent change in the breathing-related discharge activity in HMNs, which was in the opposite direction to that elicited by H1152 ejection. Interestingly, we also have recently reported that LPA, acting via the GPCR LPA_1_, regulates MN IME in a TASK1-dependent manner (Gento-Caro et al., 2021a,b). As we reaffirm in this work, sLPA-induced increment in intrinsic excitability was evident and reversible upon pharmacological inhibition of ROCK in HMNs from *wt* and *task3*^–/–^, but absent in *task1*^–/–^ mice. The fact that either inhibition of G_αi/o_ protein (PTX) or pre-treatment with siRNA*_*rock*2_*, but not with siRNA*_*rock*1_*, avoided both sLPA-induced stimulation of ROCK and IME increase in SMNs, strongly support that the mechanism by which sLPA impacts MN IME involves G_αi/o_/ROCK2 signaling. On the basis of the results from our preceding report (Gento-Caro et al., 2021a,b), together with those obtained from the current work, we propose a mechanism by which bioactive lysophospholipids modulate MN IME by recruiting at least LPA_1_/G_αi/o_/ROCK2/TASK1 signaling (Figure 8). Given widespread expression of the components of this pathway all along the nervous system, it is foreseen that this signaling cascade might fine-tune excitability in a number of neuronal populations and networks.

Several neurotransmitters, such as 5-HT, norepinephrine, substance P, TRH, and glutamate, acting on G_αq_-protein-coupled receptors increase intrinsic excitability in MNs by inhibition of TASK1 subunits (Talley et al., 2000; Chen et al., 2006). Mechanism downstream activation of metabotropic receptors by these ligands relies on direct interaction of G_αq_ with the ion channel or a closely associated intermediary (Chen et al., 2006). Here, we contribute strong evidence that this associated intermediary could be ROCK2. In this line, TRH and 5-HT-induced inward shifts in I_holding_ of HMNs were drastically reduced by G_αq_ and ROCK inhibitors. Both GPCR ligands evoked stimulation of ROCK by a G_αq_-dependent mechanism in the HN, which shares similarities with what is involved in eliciting LPA-mediated sustained contraction of gastric smooth muscle (Sriwai et al., 2008). It was remarkable that after ROCK2 knockdown, TRH and 5-HT induced in SMNs an unexpected outward shift of I_holding_. This agrees with the emergence or unmasking of a neurotransmitter-triggered mechanism increasing background resting currents. In this context, other kinases of the AGC family, PKA and PKG, which can modulate TASK1 channels (Olschewski et al., 2006; Xu et al., 2006; Toyoda et al., 2008, 2010), are profusely expressed in MNs, which impact on inspiratory drive current in HMNs, and are candidates for mediating changes in excitability induced by various neuromodulators (Feldman et al., 2005). In particular, PKG activation or inhibition potentiates or depresses, respectively, TASK1-mediated currents (Toyoda et al., 2008, 2010). On the other hand, PKA mediates adenosine-induced TASK1 inhibition in type I cells of rat carotid bodies (Xu et al., 2006) and TASK1 opening in human pulmonary artery smooth muscle cells (Olschewski et al., 2006). Interestingly, TRH and 5-HT induce activation of PKA in different neuronal types (Luo and Stopa, 2004; Tanaka et al., 2019). Thus, whether modulation of TASK1 by neurotransmitters relies on the opposite and unbalanced action of ROCK2 over other kinases such as PKA/PKG is a matter that merit further and specific research. In line with this possibility, a reciprocal regulation of MYPT1 phosphorylation by ROCK and PKA occurs downstream the G_αq_-protein-coupled receptor LPA_3_ in the gastric smooth muscle (Sriwai et al., 2008). It was also remarkable that whilst ROCK1 knockdown did not affect TRH and 5-HT-induced increase of ROCK activity in SMNs, transmitter-induced inward shift of I_holding_ in these cells was even highlighted in comparison to the control cRNA-treated group. These outcomes suggest that although ROCK1 does not contribute meaningfully to baseline ROCK activity in SMNs, basal expression levels of this isoform condition the action of ROCK2 as mediator of the TRH and 5-HT effects on background conductance, mainly due to TASK1 channel inhibition (Talley et al., 2000; Chen et al., 2006). Thus, a new research line remains open on the role and mechanism by which ROCK1 impacts TASK1-mediated current in MNs. It is of interest that ROCK1 knockdown did not emphasize sLPA-evoked changes in SMNs IME, maybe indicating subtle differences in triggered mechanisms downstream G_αq_ and G_αi/o_ proteins.

Given that ROCK activity is modulated by glutamate acting throughout ionotropic (AMPAR/NMDAR) and metabotropic (mGLUR1) receptors (Jeon et al., 2002; Kim et al., 2004; Sladojevic et al., 2020) and by another neurotransmitters systems (TRH and 5-HT) and neuromodulators (LPA) acting via GPCRs, we identify here a further partner in mediating adjustment of neuronal excitability with basic and clinical relevance. TASK1 currents can be modulated by multiple neurotransmitter systems, including those associated with awakening and alertness states, and thus, the mechanism we propose here (Figure 8) might underline coupling of neuronal responsiveness to afferent drive and behavioral status (McCormick and Bal, 1997; Talley et al., 2000; Bayliss et al., 2003).

## Data Availability Statement

The original contributions presented in the study are included in the article/Supplementary Material, further inquiries can be directed to the corresponding author/s.

## Ethics Statement

The animal study was reviewed and approved by Local Animal Care and Ethics Committee (University of Cádiz, Cádiz, Spain) and the Ministry of Agriculture, Fisheries and Rural Development (Junta de Andalucía, Spain).

## Author Contributions

BM-L conceptualized, designed the study, and wrote the manuscript. DG-F supervised electrophysiological experiments and reviewed the first draft of the manuscript. VG-M, ÁG-C, FP, and FM performed the experiments, data analysis, and the figures. BM-L and DG-F obtained the funding. All authors participated in the critical review of the manuscript.

## Conflict of Interest

The authors declare that the research was conducted in the absence of any commercial or financial relationships that could be construed as a potential conflict of interest.

## Publisher’s Note

All claims expressed in this article are solely those of the authors and do not necessarily represent those of their affiliated organizations, or those of the publisher, the editors and the reviewers. Any product that may be evaluated in this article, or claim that may be made by its manufacturer, is not guaranteed or endorsed by the publisher.

## Footnotes

1
https://portal.brain-map.org/

## Supplementary Material

Supplementary Figure 1 *Rock2* mRNA is more abundant than *rock1* mRNA in SMNs. Expression levels of mRNA for the indicated ROCK isoforms obtained by *q*RT-PCR in SMN cultures at 6 days after planting (DIV) relative to the housekeeping *gapdh*. *N* = 6 assays. Error bars, SEM. ^∗∗^*p* < 0.01; by non-parametric Mann-Whitney *U*-test.

Supplementary Figure 2 Comparative analysis of the efficiency and specificity of siRNAs to reduce mRNAs for ROCK in SMNs. **(A–D)** Expression levels of mRNAs for both ROCK isoforms obtained by *q*RT-PCR in SMN cultures at 6 DIV. SMNs received the indicated oligonucleotides, delivered by Dharmacon (numbered 1 to 4), from 2 DIV. Boxed areas signal the specific oligonucleotides selected for use in all further experiments. Selection of siRNA was based on its ability to efficiently reduce the expression of the appropriated mRNA without affecting mRNA expression of the other isoform. Sequences of the selected siRNAs are stated in Supplementary Table 1. *gapdh* was the housekeeping gene. *n* = 6 assays per condition except for *rock2* mRNA/siRNA*_*rock*1_*(2) (*n* = 4 assays). Error bars, SEM. ^∗^*p* < 0.05, ^∗∗^*p* < 0.01, ^∗∗∗^*p* < 0.001; ns, not significant; by one-way ANOVA with *post hoc* Holm-Sidak method relative to cRNA.

Supplementary Figure 3 sLPA-induced increase of HMN IME is mediated by its binding to G_αi/o_-protein-coupled receptors. Mean values of Vm (top) and R_N_ (bottom) for neonatal (P7-P9) rat HMNs recorded with an internal pipette solution containing the G_αi/o_ protein inhibitor PTX (100 ng/ml) and measured sequentially under each indicated condition. *n* = 5 HMNs. Error bars, SEM. ns, not significant; by one-way repeated measures ANOVA with *post hoc* Holm-Sidak method.

Supplementary Figure 4 ROCK knockdown does not alter SMN IME *per se*. **(A)** Top, voltage responses to a series of hyperpolarizing current pulses (0.5 s duration, 0.02 nA increments) obtained in three SMNs at 6 DIV pre-treated with indicated oligonucleotides from 2 DIV. Bottom, currents evoked by ramping the membrane potential from –120 to –40 mV (40 mV/s) plotted against holding potential (Vh) at stated conditions indicated by color code. **(B)** Mean values of Vm, R_N_, and G_*s*_ displayed by SMNs treated as in **(A)**. cRNA, *n* = 6 SMNs; siRNA*_*rock2*_*, *n* = 7 SMNs; siRNA*_*rock1*_*, *n* = 6 SMNs. Error bars, SEM. ns, not significant; by one-way ANOVA with *post hoc* Holm-Sidak method relative to the cRNA.
