# Supplementary figures and images for: Lysophosphatidic Acid and Several Neurotransmitters Converge on Rho-Kinase 2 Signaling to Manage Motoneuron Excitability
Source: Front Mol Neurosci. 2021 Dec 6;14:788039. doi: 10.3389/fnmol.2021.788039 (PMC8685439; doi:10.3389/fnmol.2021.788039)

Supplementary Figure 1

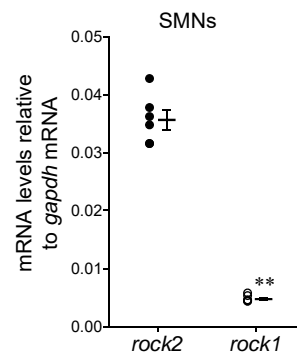

Supplementary Figure 2

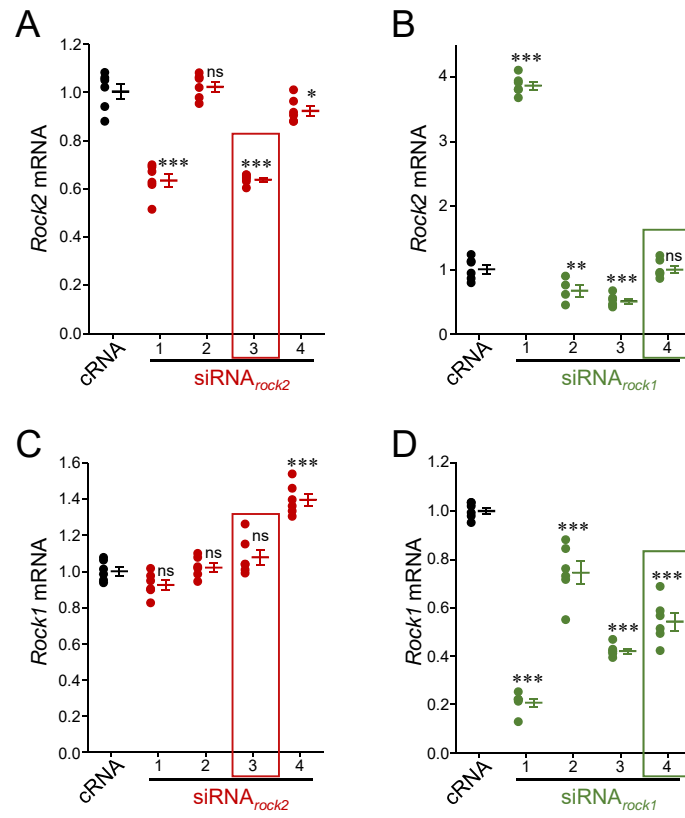

Supplementary Figure 3

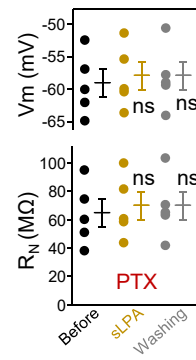

## Supplementary Figure 4

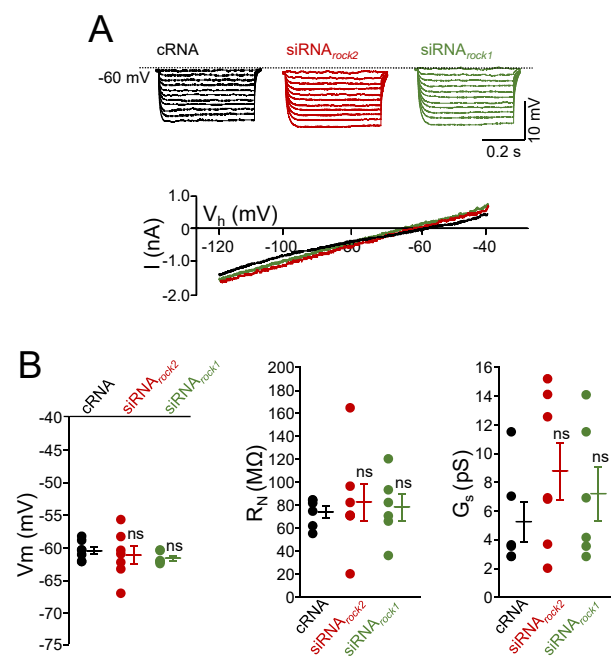

Supplement: Supplementary Figure 1 — Rock2 mRNA is more abundant than rock1 mRNA in SMNs. Expression levels of mRNA for the indicated ROCK isoforms obtained by qRT-PCR in SMN cultures at 6 days after planting (DIV) relative to the housekeeping gapdh. N = 6 assays. Error bars, SEM. ∗∗p < 0.01; by non-parametric Mann-Whitney U-test. [file Data_Sheet_1.pdf]
