# Supplementary material for: Lysophosphatidic Acid and Several Neurotransmitters Converge on Rho-Kinase 2 Signaling to Manage Motoneuron Excitability
Source: Front Mol Neurosci. 2021 Dec 6;14:788039. doi: 10.3389/fnmol.2021.788039 (PMC8685439; doi:10.3389/fnmol.2021.788039)
Supplement: Supplementary file 2 [file Data_Sheet_2.PDF]

Table S1.- Primers for *q*RT-PCR analysis and siRNAs for ROCK knockdown.

Oligonucleotide primer sequences (5' → 3')

Gene	Forward	Reverse
For mice		
rock1	CCAGAATCACAAAGGCCATGA	TCTGTGGCACTTAACATGGCA
rock2	ATTGAACAGCTGCGGTCACAG	GCACAGGCAATGACAACCAT
gapdh	AGAACATCATCCCTGCATCCA	AGATCCACGACGGACACATTG
For rats		
rock1	TGCGGGAGTTACAAGATCAGCT	TTTCCGTCAGTCATCAGCAC
rock2	CGAATAGAACTCCAGATGACC	GCACAGGCAATGACAACC
gapdh	AGAACATCATCCCTGCATCCA	AGATCCACGACGGACACATTG

siRNA sequences (5' → 3') for ROCK knockdown

$siRNA_{rock1}$	UGUUGUUCUAUAAUGAUGA
$siRNA_{rock2}$	CUUUAGAGAAAAUUUGUUA

Table S2.- Membrane parameters of rat HMNs under the indicated experimental conditions.

	Vm (mV)	R_N (MΩ)	I_{th} (nA)	G_s (pS)	G_N (pS)
**Control**	-60.9 ± 0.8 (17)	68.4 ± 4.4 (15)	0.64 ± 0.05 (17)	12.1 ± 1.2 (5)	12.9 ± 1.2 (5)
**H1152**	-60.9 ± 1.2 (5)	69.5 ± 4.9 (5)	0.77 ± 0.02 (5)	14.1 ± 0.5 (5)	14.4 ± 0.7 (5)
**aROCK2**	-53.9 ± 3.6 (5)*	92.8 ± 5.6 (5)*	0.26 ± 0.07 (5)**	8.4 ± 0.7 (7)***	9.3 ± 0.6 (5)***
**aROCK2 +H1152**	-66. 6 ± 1.6 (5)	61.5 ± 8.1 (5)	0.75 ± 0.01 (5)	14.9 ± 0.5 (7)	13.4 ± 0.6 (5)

Number of analyzed HMNs per condition is indicated in parentheses. * $p < 0.05$; ** $p < 0.01$; *** $p < 0.001$, by one-way ANOVA with *post hoc* Holm-Sidak method relative to the control pool.

Table S3.- H1152-induced changes in membrane parameters of rat HMNs.

	**ΔVm (mV)**	**ΔR_N (MΩ)**	**ΔI_{th} (nA)**	**ΔG_{slope} (pS)**	**ΔG_N (pS)**
**Untreated**	1.1 ± 1.2 (5)	3.4 ± 3.6 (5)	0.03 ± 0.06 (5)	-2.0 ± 0.7 (5)	-1.5 ± 0.6 (5)
**aROCK2**	-12.6 ± 3.2 (5)*	31.3 ± 5.5 (5)**	0.49 ± 0.13 (5)*	5.9 ± 0.5 (5)*	4.2 ± 0.2 (5)**

Number of analyzed HMNs is indicated in parentheses. * $p < 0.05$; ** $p < 0.01$; *** $p < 0.001$, by unpaired Student *t*-test relative to untreated HMNs.

Table S4.- Membrane parameters of mouse HMNs with indicated genotype and under stated conditions.

	Vm (mV)	R_N (MΩ)	I_{th} (nA)	G_s (pS)
		wt		
**Control**	-61.8 ± 0.9 (6)	76.5 ± 4.5 (6)	0. 69 ± 0.05 (6)	13.7 ± 0.6 (6)
**H1152**	-62.1 ± 0.9 (6)	75.0 ± 3.7 (6)	0. 66 ± 0.04 (6)	13.2 ± 0.4 (6)
**aROCK2**	-53.5 ± 3.0 (7)**	100.0 ± 7.3 (7)*	0.59 ± 0.07 (6)	8.1 ± 0.7 (6)*
**aROCK2 +H1152**	-61.9 ± 0.7 (7)	76.9 ± 3.0 (7)	0.71 ± 0.06 (6)	15.2 ± 0.7 (6)
		task1 $^{-/-}$		
**Control**	-62.1 ± 0.8 (5)	77.0 ± 4.5 (5)	0.77 ± 0.03 (5)	13.4 ± 0.7 (5)
**H1152**	-62.2 ± 1.1 (5)	76.7 ± 4.7 (5)	0.73 ± 0.04 (5)	13.5 ± 0.7 (5)
**aROCK2**	-59.4 ± 1.7 (6)	75.2 ± 5.9 (6)	0.59 ± 0.08 (6)	14.5 ± 0.5 (5)
**aROCK2 +H1152**	-62.7 ± 2.0 (6)	77.0 ± 6.7 (6)	0.53 ± 0.07 (6)	15.4 ± 0.8 (5)
		task3 $^{-/-}$		
**Control**	-62.1 ± 0.8 (7)	78.2 ± 3.4 (7)	0.78 ± 0.05 (7)	12.6 ± 0.5 (7)
**H1152**	-62.5 ± 0.7 (7)	72.7 ± 4.5 (7)	0.77 ± 0.06 (7)	12.7 ± 0.6 (7)
**aROCK2**	-50.4 ± 1.3 (7)*	94.9 ± 4.9 (7)*	0. 62 ± 0.06 (7)*	10.6 ± 1.1 (6)*
**aROCK2 +H1152**	-61.6 ± 1.2 (7)	77.6 ± 11.8 (7)	0.95 ± 0.05 (7)	16.5 ± 0.9 (6)

Number of analyzed HMNs per condition is indicated in parentheses. * $p < 0.05$; ** $p < 0.01$; *** $p < 0.001$, by one-way ANOVA with *post hoc* Holm-Sidak method relative to the control pool for each genotype.

Table S5.- H1152-induced changes in membrane parameters of mouse HMNs with indicated genotypes and under stated conditions.

	**ΔVm (mV)**	**ΔR_N (MΩ)**	**ΔI_{th} (nA)**	**ΔG_s (pS)**
		wt		
**Untreated**	-0.3 ± 0.4 (6)	-1.6 ± 1.2 (6)	-0.02 ± 0.02 (6)	-0.5 ± 0.3 (6)
**aROCK2**	8.4 ± 2.6 (7)*	23.1 ± 6.1 (7)*	-0.12 ± 0.03 (7)*	-7.0 ± 1.1 (7)*
		task1$^{-/-}$		
**Untreated**	0.0 ± 0.9 (6)	-0.3 ± 1.4 (6)	-0.04 ± 0.02 (6)	0.1 ± 0.6 (6)
**aROCK2**	3.3 ± 0.5 (6)	-1.8 ± 1.2 (6)	0.06 ± 0.03 (6)	-0.9 ± 0.4 (6)
		task3$^{-/-}$		
**Untreated**	-0.4 ± 0.6 (7)	-5.6 ± 4.5 (7)	-0.01 ± 0.02 (7)	0.1 ± 0.5 (7)
**aROCK2**	11.2 ± 1.6 (7)*	17.3 ± 5.5 (7)*	-0.33 ± 0.05 (7)*	-5.1 ± 1.1 (6)*

Number of analyzed HMNs per condition is indicated in parentheses. * $p < 0.05$; ** $p < 0.01$; *** $p < 0.001$, by unpaired Student *t*-test relative to untreated HMNs for each genotype.

Table S6.- Effects of sLPA on membrane parameters of rat HMNs subjected to indicated conditions.

	**Vm (mV)**	**R_N (MΩ)**	**I_{th} (nA)**
**Before**	-60.9 ± 0.8 (17)	68.4 ± 4.1 (17)	0. 64 ± 0.05 (17)
**sLPA**	-49.3 ± 2.4 (8)*	100.9 ± 9.8 (10)*	0.26 ± 0.06 (8)*
**sLPA+H1152**	-59.9 ± 0.9 (6)	66.4 ± 4.3 (6)	0.15 ± 0.01 (6)*
		**PTX**	
**Before**	-59.1 ± 2.1 (5)	65.1 ± 6.3 (5)	0.86 ± 0.12 (5)
**sLPA**	-58.0 ± 2.1 (5)	65.4 ± 5.8 (5)	0.81 ± 0.12 (5)
**Washing**	-57.6 ± 2.2 (5)	63.5 ± 6.3 (5)	0.81 ± 0.10 (5)

Number of analyzed HMNs per condition is indicated in parentheses. * $p < 0.05$; ** $p < 0.01$; *** $p < 0.001$, by one-way RM ANOVA relative to the before condition.

Table S7.- Effect of sLPA on membrane parameters of mouse HMNs with indicated genotypes and subjected to stated treatments.

	**Vm (mV)**	**R_N (MΩ)**	**I_{th} (nA)**	**G_S (pS)**
		wt		
**Before**	-64.8 ± 1.8 (7)	82.7 ± 4.7 (7)	0.87 ± 0.08 (7)	15.4 ± 0.9 (7)
**sLPA**	-54.3 ± 1.9 (7)*	117.6 ± 4.9 (7)*	0.33 ± 0.29 (7)*	9.6 ± 0.4 (7)*
**sLPA+H1152**	-64.2 ± 2.2 (7)	76.2 ± 5.1 (7)	0.35 ± 0.03 (7)*	13.8 ± 0.8 (7)
		task1$^{-/-}$		
**Before**	-62.1 ± 0.8 (7)	67.7 ± 10.8 (7)	0. 67 ± 0.11 (7)	15.9 ± 0.9 (7)
**sLPA**	-63.3 ± 0.7 (7)	63.6 ± 10.3 (7)	0. 63 ± 0.10 (7)	16.3 ± 0.9 (7)
**sLPA+H1152**	-63.4 ± 0.6 (7)	66.5 ± 9.4 (7)	0. 65 ± 0.09 (7)	15.8 ± 0.8 (7)
		task3$^{-/-}$		
**Before**	-64.1 ± 0.7 (11)	83.5 ± 6.6 (11)	0.47 ± 0.06 (11)	11.4 ± 0.9 (11)
**sLPA**	-55.3 ± 0.5 (11)*	100.1 ± 8.3 (11)*	0.26 ± 0.06 (11)*	8.9 ± 0.7 (11)*
**sLPA+H1152**	-63.8 ± 0.8 (11)	84.3 ± 6.3 (11)	0.39 ± 0.04 (11)	11.2 ± 1.0 (11)

Number of analyzed HMNs per condition is indicated in parentheses. * $p < 0.05$; ** $p < 0.01$; *** $p < 0.001$, by one-way RM ANOVA relative to the before condition for each genotype.

Table S8.- sLPA-induced changes in membrane parameters of SMNs pre-incubated with indicated oligonucleotides.

	Vm (mV)	I_{th} (nA)	G_S (pS)
**cRNA**			
**Before**	-60.5 ± 0.6 (7)	0.59 ± 0.09 (6)	5.2 ± 1.4 (6)
**sLPA**	-51.3 ± 2.2 (6)*	0.22 ± 0.03 (6)*	2.1 ± 1.0 (6)*
**sLPA+H1152**	-61.8 ± 0.3 (6)	0.56 ± 0.08 (6)	6.4 ± 1.7 (6)
**siRNA*rock2***			
**Before**	-61.2 ± 1.4 (7)	0.46 ± 0.09 (7)	8.8 ± 2.0 (7)
**sLPA**	-61.0 ± 1.0 (7)^{ns}	0.47 ± 0.09 (7)^{ns}	8.5 ± 1.8 (7)^{ns}
**sLPA+H1152**	-59.0 ± 0.0 (1)	na	6.8 ± 0.0 (1)
**siRNA*rock1***			
**Before**	-61.7 ± 0.4 (6)	0.63 ± 0.09 (6)	7.2 ± 1.9 (6)
**sLPA**	-50.6 ± 1.0 (6)*	0.46 ± 0.09 (6)*	2.3 ± 1.0 (6)*
**sLPA+H1152**	-60.1 ± 0.8 (5)	0.35 ± 0.04 (5) *	6.1 ± 1.3 (5)

Number of analyzed SMNs per condition is indicated in parentheses. * $p < 0.05$; ** $p < 0.01$; *** $p < 0.001$; ns, non-significant, by one-way RM ANOVA relative to the before condition for each oligonucleotide. na, non-available

Table S9.- sLPA-induced changes in ROCK activity (in percent) of SMNs pre-treated with stated oligonucleotides.

	**cRNA**	**siRNA*rock2***	**siRNA*rock1***
**None**	100.0 ± 1.0 (3)	58.7 ± 1.6 (6)***	98.0 ± 0.7 (3)
**sLPA**	198.0 ± 1.6 (3)	109.5 ± 2.1 (3)***	209.2 ± 1.0 (3)**
**sLPA+PTX**	97.2 ± 1.0 (3)	95.5 ± 1.2 (3)	96.9 ± 1.0 (3)
**sLPA+H1152**	95.3 ± 1.3 (3)	100.0 ± 1.5 (3)	91.6 ± 1.2 (3)

Number of assays per condition is indicated in parentheses. * $p < 0.05$; ** $p < 0.01$; *** $p < 0.001$; by non-parametric Mann-Whitney *U* test relative to cRNA condition for each treatment.

Table S10.- Neurotransmitter-induced changes in $I_{holding}$ (pA) of rat HMNs.

	**Before**	**TRH**	**5-HT**
**None**	-240.6 ± 40.66 (19)	-815.9 ± 74.0 (8)*	-537.4 ± 21.0 (11)*
**+YM-254890**	-312.4 ± 75.35 (7)	-382.8 ± 16.8 (7)	-353.0 ± 3.4 (9)
**+H1152**	-377.0 ± 87.82 (8)	-513.4 ± 26.3 (8)*	-413.3 ± 5.2 (8)*

Number of analyzed HMNs per condition is indicated in parentheses. * $p < 0.05$; ** $p < 0.01$; *** $p < 0.001$, by one-way ANOVA with *post hoc* Holm-Sidak method relative to the before condition.

Table S11.- Neurotransmitter-induced changes in ROCK activity (in percent) of microdissected HNs from neonatal rats.

	**Control**	**TRH**	**5-HT**
**None**	100.0 ± 1.6 (4)	253.5 ± 0.2 (4)*	260.1 ± 3.3 (4)*
**+YM-254890**	66.2 ± 0.5 (4)#	77.6 ± 1.9 (4)#	70.1 ± 0.4 (4)#

Number of assays per condition is indicated in parentheses. *,# $p < 0.05$, by non-parametric Mann-Whitney *U* test relative to the control or none condition, respectively .

Table S12.- Neurotransmitter-induced changes in ROCK activity (in percent) of SMNs pretreated with stated oligonucleotides.

	**cRNA**	**siRNA*rock2***	**siRNA*rock1***
**None**	100.0 ± 1.0 (3)	58.7 ± 1.6 (6)	98.0 ± 0.7 (3)
**TRH**	246.9 ± 5.8 (3)*	118.4 ± 3.6 (3)	262.6 ± 3.4 (3)*
**5-HT**	200.6 ± 3.7 (3)*	97.2 ± 1.0 (3)	200.3 ± 1.2 (3)*

Number of analyzed SMNs per condition is indicated in parentheses. * $p < 0.05$; ** $p < 0.01$; *** $p < 0.001$, by non-parametric Mann Whitney *U* test relative to untreated condition for each oligonucleotide.
